# Age‐stratified effectiveness of a nurse‐led heart failure programme: A population‐based study

**DOI:** 10.1111/joim.70136

**Published:** 2026-07-12

**Authors:** Blanca Torres‐Cardús, Emili Vela‐Vallespín, David Monterde, Gerard Carot‐Sans, Jordi Piera‐Jiménez, Júlia Folguera, Laia Alcober‐Morte, Raúl Ramos‐Polo, Encarnación Hidalgo‐Quirós, Núria José‐Bazán, Sergi Yun Viladomat, Pedro Moliner, Santiago Jiménez‐Marrero, Alberto Garay Melero, Alexandra Pons Riverola, Lídia Alcoberro Torres, Herminio Morillas, Betlem Salvador‐González, Sílvia Jovells‐Vaqué, Mireia Andrés Villareal, Cristina Enjuanes, Josep Comin‐Colet

**Affiliations:** ^1^ Primary Care Service Delta del Llobregat South Metropolitan Territory Management, Catalan Institute of Health L'Hospitalet de Llobregat Spain; ^2^ MARCEVAP Research Group IDIAP Jordi Gol Barcelona Spain; ^3^ Faculty of Medicine and Health Sciences Universitat de Barcelona (UB) Barcelona Spain; ^4^ Catalan Health Service Barcelona Spain; ^5^ Digitalization for the Sustainability of the Healthcare System DS3‐IDIBELL Bellvitge Institute for Biomedical Research L'Hospitalet de Llobregat Spain; ^6^ Faculty of Informatics Telecommunications and Multimedia Universitat Oberta de Catalunya Barcelona Spain; ^7^ CIBERCV Research Group Centro de Investigación Biomédica en Red de Enfermedades Cardiovasculares Madrid Spain; ^8^ Community Heart Failure Program Cardiology Department Bellvitge University Hospital L'Hospitalet de Llobregat Barcelona Spain; ^9^ Bio‐Heart Cardiovascular Diseases Research Group Bellvitge Biomedical Research Institute (IDIBELL) L'Hospitalet de Llobregat Barcelona Spain; ^10^ South Metropolitan Primary Care Research Support Unit Catalan Health Institute L'Hospitalet de Llobregat Barcelona Spain; ^11^ Heart Institute of Bellvitge University Hospital L'Hospitalet de Llobregat Barcelona Spain; ^12^ Directorate of Innovation, Investigation and Universities, Southern Metropolitan Area Catalan Health Institute L'Hospitalet de Llobregat Barcelona Spain

**Keywords:** aged, chronic heart failure, heart failure (HF) management programmes, mortality, vulnerable populations

## Abstract

**Aim:**

Old age is associated with poor outcomes in patients with heart failure (HF). This study aimed to assess the impact of age on the effectiveness of a new integrated, transition‐based nurse‐led HF programme in a healthcare area with 209,255 inhabitants.

**Methods:**

We conducted a population‐based evaluation of all patients discharged from the hospital with International Classification of Diseases, ninth revision, Clinical Modification code for HF as the primary diagnosis in Catalonia between 2015 and 2019. We compared outcomes between patients exposed to the new HF programme with the rest of Catalonia, stratified by age.

**Results:**

A total of 77,554 patients with HF were classified into three groups: 15–74, 75–84 and >84 years. In the Bellvitge University Hospital (HUB)‐Delta area (3396 HF patients), comparison of the pre‐implementation and consolidation periods of the programme showed a decrease in clinically related hospitalisation in the youngest group (hazard ratio [HR] 0.87; confidence interval [CI] 0.84–0.91, *p *< 0.001), the middle‐age group (HR 0.90; CI 0.87–0.93, *p* < 0.001) and the oldest group (HR 0.83; CI 0.83–0.89; *p *< 0.001). Similar results were observed for all‐cause mortality and readmission. Compared with HF care in the rest of Catalonia, the new programme resulted in a significant reduction in all‐cause mortality across all age groups and all‐cause hospitalisations among the young and middle‐aged groups during the programme's transition and consolidation periods.

**Conclusions:**

Implementation of the new HF programme within a universal‐coverage healthcare system was associated with significant reductions in all‐cause clinically related hospitalisations and HF readmissions across all age groups.

## Graphical abstract

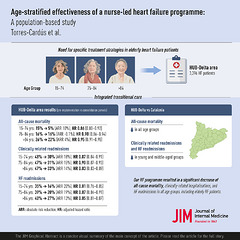

**Abbreviations**
CKD: chronic kidney disease
COPD: chronic obstructive pulmonary disease
GMA: adjusted morbidity groups
HF: heart failure
HUB: Bellvitge University Hospital
HUB‐Delta: Bellvitge University Hospital (HUB) and Delta Primary Care Service
ICD‐9‐CM: International Classification of Diseases, ninth revision, Clinical Modification
IDIBELL: Bellvitge Biomedical Research Institute
MI: myocardial infarction

## Introduction

Heart failure (HF) is a fatal disease and a substantial health concern owing to its high rates of morbidity and mortality and reduced health‐related quality of life. As the worldwide population ages and treatment strategies improve, the prevalence of HF is increasing, especially among older populations [1, 2, 3]. Advanced age has been previously associated with poorer outcomes in HF, with increased event rates and a higher prevalence of comorbidities requiring greater allocation of resources and placing increasing pressure on healthcare systems [4, 5, 6, 7, 8, 9, 10].

In recent decades, advances in pharmacological treatments and the introduction of HF programmes have shifted the course of the disease towards more favourable outcomes and lower healthcare costs. Disease management programmes accompany patients throughout their entire care pathway, from diagnosis through acute events to terminal stages. They aim to optimise lifestyle management, pharmacological evidence‐based treatments and device therapy, promote education and self‐care, and provide access to healthcare when needed [11, 12, 13, 14, 15, 16, 17].

Many studies have addressed and demonstrated the effectiveness of HF management programmes; however, this effectiveness has not been independently proven when evaluated according to age.

Some authors have focused on better characterising older patients with HF, describing their sociodemographic data and comorbidities, mostly in population‐based studies from national registries [4, 5, 6, 7, 8, 9, 10]. Others have attempted to determine the effect of HF programmes and age through meta‐analyses, including trials with high heterogeneity and lack of specification of the interventions and through clinical trials that did not represent the real‐world population because of the inherent selection bias [18, 19, 20]. These studies, which included a small number of patients, were not stratified by age, reported surprisingly low mean ages or had exclusion criteria that included typical age‐related comorbidities [21, 22, 23, 24, 25, 26, 27].

Therefore, evidence of the effectiveness of HF programmes according to age, especially in older adults in real‐world settings, remains scarce.

Considering these limitations, our study was designed to assess the impact of age on the effectiveness of a multidisciplinary HF programme for patients recently discharged after hospitalisation for HF.

To address this objective, we first implemented a transitional, nurse‐led, multidisciplinary HF programme in a healthcare area and then evaluated the influence of age on the effectiveness of the new programme in terms of clinically related hospitalisations, HF readmissions and all‐cause mortality.

## Methods

### Study setting

The study was conducted in Catalonia (*N* = 7,816,419 in 2019), where universal public healthcare coverage is guaranteed by law. Recently, the Catalan Health Service has promoted several quality‐of‐care improvement initiatives for patients with chronic HF [12, 16, 21].

In 2017, a new programme to improve the quality of care for patients with chronic HF was developed and implemented in the Bellvitge University Hospital (HUB) and Delta Primary Care Service (HUB‐Delta) integrated healthcare area (209,255 inhabitants). The HUB‐Delta HF programme was designed as a multidisciplinary, transitional‐care and nurse‐led programme based on the conceptual framework provided by the Chronic Care Model, which includes all components of care and interventions that have shown benefits in patients with HF [13, 15, 16, 17, 28, 29]. This model, which was previously successfully implemented in a different healthcare area, was further refined in the current implementation. The characteristics of the HUB‐Delta HF programme are described in Section SA.

### Data sources and data quality control

Since 2011, the Health Department of the Government of Catalonia has used an automated administrative healthcare database, the Catalan Health Surveillance System [1, 3, 30, 31, 32, 33].

This database integrates information from several sources, including the vital status of citizens (Spanish National Statistics Institute). Further details are provided in the Section SB.

### Study design, study population, coding criteria and ethics

The study design has been described previously [34]. To evaluate the influence of age on the effectiveness of the new HF programme, we followed three steps.

First, we designed and implemented a comprehensive HF programme in the HUB‐Delta healthcare area between November 2016 and December 2019. We considered three different periods: the pre‐implementation period (2015–2016), the transition period (2017) and the consolidation period (2018–2019).

Second, we designed a pragmatic population‐based evaluation of the implementation of the programme by conducting a natural experiment. For this study, we included all individuals consecutively admitted to the hospital with at least one International Classification of Diseases, ninth revision, Clinical Modification (ICD‐9‐CM) code for HF as the primary diagnosis in Catalonia between 1 January 2015 and 31 December 2019. Only patients discharged alive were included in the analysis. The ICD‐9‐CM codes used are described in the Sections C and F.

Data on clinical characteristics and previous medical resource use were obtained at baseline for all patients. Clinical outcomes (clinically related hospitalisations, HF readmissions and all‐cause mortality) were measured and analysed for all patients between 2015 and 2019 across predetermined age groups: younger (15–74 years), middle‐aged (75–84 years) and older (>84 years) groups.

In the third step, we measured the effectiveness of the programme implementation across age strata at two levels: first, within‐group comparison, assessing the differences across implementation periods in the HUB‐Delta area, taking the year 2015 (pre‐implementation) and the period 2015–2016 as a reference, and second, between‐group comparison, contrasting outcomes between patients in the HUB‐Delta area and patients in the rest of Catalonia at each predefined period.

For descriptive analyses, the absolute risk reduction (ARR) was calculated as the absolute difference in crude event proportions between the pre‐implementation (2015–2016) and consolidation (2018–2019) periods in both healthcare areas.

Further details regarding the assessment of covariates, quality‐of‐care indicators and study endpoints are provided in the Section SD.

This study was conducted in accordance with the principles of the Declaration of Helsinki. All data were handled according to the General Data Protection Regulation 2016/679 on data protection and privacy for all individuals within the European Union and the local regulatory framework regarding data protection. The independent ethics committee of HUB and Bellvitge Biomedical Research Institute approved the study protocol and waived the requirement for informed consent for the use of healthcare data (reference number PR029/24).

### Outcomes

The primary outcome variable was time to the first clinically related readmission after discharge from the index hospitalisation for HF. Clinically related readmissions included all hospital admissions attributable to HF or common comorbidities (Section SD).

Secondary outcome variables included the time to the first HF readmission, defined as the first hospitalisation with HF as the primary diagnosis after discharge, and the time to death from any cause, measured from the date of discharge after the index HF hospitalisation.

All outcomes were assessed across the three pre‐defined age groups (15–74 years, 75–84 years and >84 years) and evaluated within the HUB‐Delta healthcare area across implementation periods and between the HUB‐Delta area and the rest of Catalonia. Further details on outcome definitions, coding criteria and data extraction procedures are provided in the Supporting Information Appendices C and D.

### Statistical analyses

Several multivariate‐adjusted Cox proportional hazards models were constructed to analyse the impact of the new HF programme at two levels: (1) before and after its implementation in our healthcare area, stratified according to age, and (2) compared with the HF population managed outside our hospital within the same period (i.e., comparison of HUB‐Delta vs. the rest of the patients with HF covered by the Catalan Health Service), stratified by age. All models were adjusted for sex, individual socioeconomic status, previous hospitalisation, time since diagnosis of HF and comorbidity burden, measured using the adjusted morbidity groups index (GMA for Catalan ‘Grups de Morbiditat Ajustada’) [35].

All statistical tests and confidence intervals (CIs) were constructed with a Type I error alpha level of 5% with no adjustments for multiplicity. Statistical significance was set at *p* < 0.05. All analyses were performed using the R software (version 4.0.2; R Foundation for Statistical Computing, Vienna, Austria). A more detailed description is presented in the Section SE.

## Results

### Study participants

**Fig. 1:**
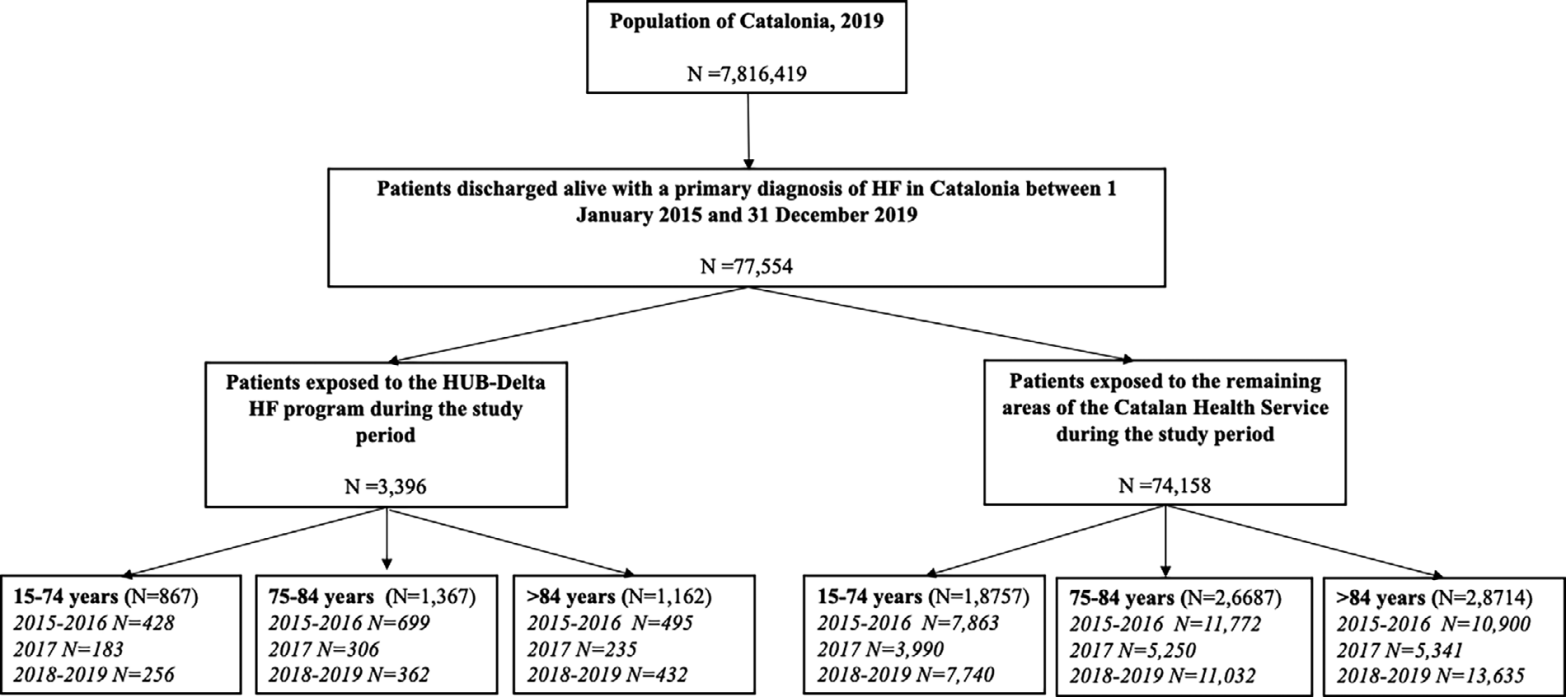
Flow diagram of patients of the study population included in the present analysis. HF, heart failure; HUB, Bellvitge University Hospital.

The study cohort consisted of 77,554 patients who were discharged alive with a diagnosis of HF between 1 January 2015 and 31 December 2019. Of these, 3396 patients were exposed to the HUB‐Delta programme, and 74,158 patients were managed in the remaining healthcare areas of Catalonia. The main results of this study have been previously published [34]. The flow chart of the patients in this study is shown in Fig. 1.

The baseline characteristics of the patients exposed to the HUB‐Delta programme are presented in Table 1. Patients were classified according to age into the 15–74 years (867; 26%), 75–84 years (1367; 40%) and >84 years (1162; 34%) age groups. The mean age was 79 years (standard deviation, 10 years), and 53% of the patients were women. The proportion of women increased with age, as did the comorbidity burden measured using the GMA index, except in the oldest group, which had a lower GMA index. Almost 80% of the patients belonged to the low socioeconomic status group. Nearly half of the patients had been diagnosed with HF for less than 1 year.

**Table 1:** Baseline characteristics according to age group strata of patients discharged alive with a primary diagnosis of HF in the Bellvitge University Hospital (HUB) and Delta Primary Care Service (HUB‐Delta) healthcare area between 1 January 2015 and 31 December 2019.

	Total	15–74 years	75–84 years	>84 years	
	*N* = 3396	*N* = 867	*N* = 1367	*N* = 1162	*p*‐value
Sex:					<0.001
Male	1599 (47%)	552 (64%)	637 (47%)	410 (35%)	
Female	1797 (53%)	315 (36%)	730 (53%)	752 (65%)	
Age	79 (10)	66 (8)	80 (3)	89 (3)	<0.001
Risk levels (GMA):					<0.001
Low or intermediate risk	626 (18%)	210 (24%)	206 (15%)	210 (18%)	
High risk	1482 (44%)	337 (39%)	594 (44%)	551 (47%)	
Very high risk	1288 (38%)	320 (37%)	567 (41%)	401 (35%)	
Morbidity burden (GMA)	37.0 (15.8)	36.0 (17.4)	38.6 (15.6)	35.9 (14.7)	<0.001
Individual annual income:					<0.001
Medium or high SES	550 (16%)	207 (24%)	217 (16%)	126 (11%)	
Low SES	2669 (79%)	579 (67%)	1081 (79%)	1009 (87%)	
Very low SES	177 (5%)	81 (9%)	69 (5%)	27 (2%)	
Years since HF diagnosis:					<0.001
<1 year	1384 (41%)	394 (45%)	531 (39%)	459 (40%)	
1–2 years	587 (17%)	126 (15%)	261 (19%)	200 (17%)	
3–5 years	692 (20%)	141 (16%)	283 (21%)	268 (23%)	
>5 years	733 (22%)	206 (24%)	292 (21%)	235 (20%)	
Previous MI	665 (20%)	204 (24%)	292 (21%)	169 (15%)	<0.001
Atrial fibrillation	1221 (36%)	298 (34%)	514 (38%)	409 (35%)	0.242
Peripheral vascular disease	930 (27%)	290 (33%)	386 (28%)	254 (22%)	<0.001
Hypertension	3095 (91%)	721 (83%)	1277 (94%)	1097 (94%)	<0.001
Obesity	1096 (32%)	378 (44%)	472 (35%)	246 (21%)	<0.001
Smoking habit	809 (24%)	361 (42%)	296 (22%)	152 (13%)	<0.001
Hyperlipidaemia	1431 (42%)	418 (48%)	600 (44%)	413 (36%)	<0.001
Diabetes mellitus	1733 (51%)	492 (57%)	740 (54%)	501 (43%)	<0.001
CKD	1901 (56%)	381 (44%)	789 (58%)	731 (63%)	<0.001
Anaemia	1840 (54%)	399 (46%)	761 (56%)	680 (59%)	<0.001
COPD	1440 (42%)	374 (43%)	631 (46%)	435 (37%)	<0.001
Cancer	575 (17%)	127 (15%)	257 (19%)	191 (16%)	0.033
Osteoarthritis	361 (11%)	87 (10%)	150 (11%)	124 (11%)	0.778
Severe cognitive impairment	194 (6%)	7 (1%)	75 (5%)	112 (10%)	<0.001
Cirrhosis	77 (2%)	42 (5%)	21 (2%)	14 (1%)	<0.001
Major mental health disorder	411 (12%)	178 (21%)	155 (11%)	78 (7%)	<0.001
Alcohol abuse	645 (19%)	285 (33%)	254 (19%)	106 (9%)	<0.001
Opioid abuse	12 (0.4%)	7 (0.8%)	4 (0.3%)	1 (0.1%)	0.029
Cocaine abuse	13 (0.4%)	11 (1.3%)	2 (0.2%)	0 (0%)	<0.001
Number of previous hospital admissions	1.2 (1.6)	1.2 (1.6)	1.3 (1.6)	1.1 (1.5)	0.026
Number of days in hospital (previous)	9.4 (15.6)	11.4 (19.1)	10.4 (16.5)	6.8 (10.3)	<0.001
Number of days in psychiatric unit (previous)	0.0 (0.9)	0.0 (1.4)	0.0 (0.2)	0.0 (0.8)	0.290
Number of days in skilled nursing facility (previous)	2.8 (18.8)	2.0 (16.6)	2.9 (20.8)	3.2 (17.9)	0.319
Number of days in nursing home (previous)	15.0 (69.6)	4.9 (39.3)	8.2 (52.0)	30.5 (97.3)	<0.001

*Note*: Continuous data are presented as mean (SD). Categorical data are presented as *n* (%).

Abbreviations: CKD, chronic kidney disease; COPD, chronic obstructive pulmonary disease; GMA, adjusted morbidity groups; HF, heart failure; HUB, Bellvitge University Hospital; MI, myocardial infarction; SES, socioeconomic status.

As shown in Table 2, the differences in the baseline characteristics of patients in the HUB‐Delta according to age groups were similar to those observed in the entire cohort. However, patients in the HUB‐Delta programme were slightly younger than those in the rest of Catalonia.

**Table 2:** Baseline characteristics, including age groups of patients discharged alive with a primary diagnosis of heart failure (HF) to healthcare areas (rest of Catalonia vs. Bellvitge University Hospital (HUB) and Delta Primary Care Service [HUB‐Delta]) between 1 January 2015 and 31 December 2019.

	Rest of Catalonia	HUB‐Delta	
	*N* = 74,158	*N* = 3396	*p*‐value
Sex			0.347
Male	34,296 (46%)	1599 (47%)	
Female	39,862 (54%)	1797 (53%)	
Age	80 (10.8)	79 (10)	0.018
Age group			<0.001
15–74 years	18,726 (25%)	867 (26%)	
75–84 years	26,687 (36%)	1367 (40%)	
>84 years	28,714 (39%)	1162 (34%)	
Morbidity burden (GMA)	37.9 (16.4)	37.0 (15.8)	0.001
Risk levels (GMA)			0.004
Low risk	1727 (2.3%)	66 (2%)	
Intermediate risk	11,628 (16%)	560 (17%)	
High risk	30,656 (41%)	1482 (44%)	
Very high risk	30,147 (41%)	1288 (38%)	
Individual annual income			0.074
Medium or high SES	11,468 (16%)	550 (16%)	
Low SES	59,298 (80%)	2669 (79%)	
Very low SES	3358 (4.5%)	177 (5%)	

*Note*: Continuous data are presented as mean (SD). Categorical data are presented as *n* (%).

Abbreviations: GMA, adjusted morbidity groups; HUB, Bellvitge University Hospital; SES, socio‐economic status.

### Effectiveness of the implementation of the HUB‐Delta HF programme across age strata and throughout the pre‐defined periods

**Fig. 2:**
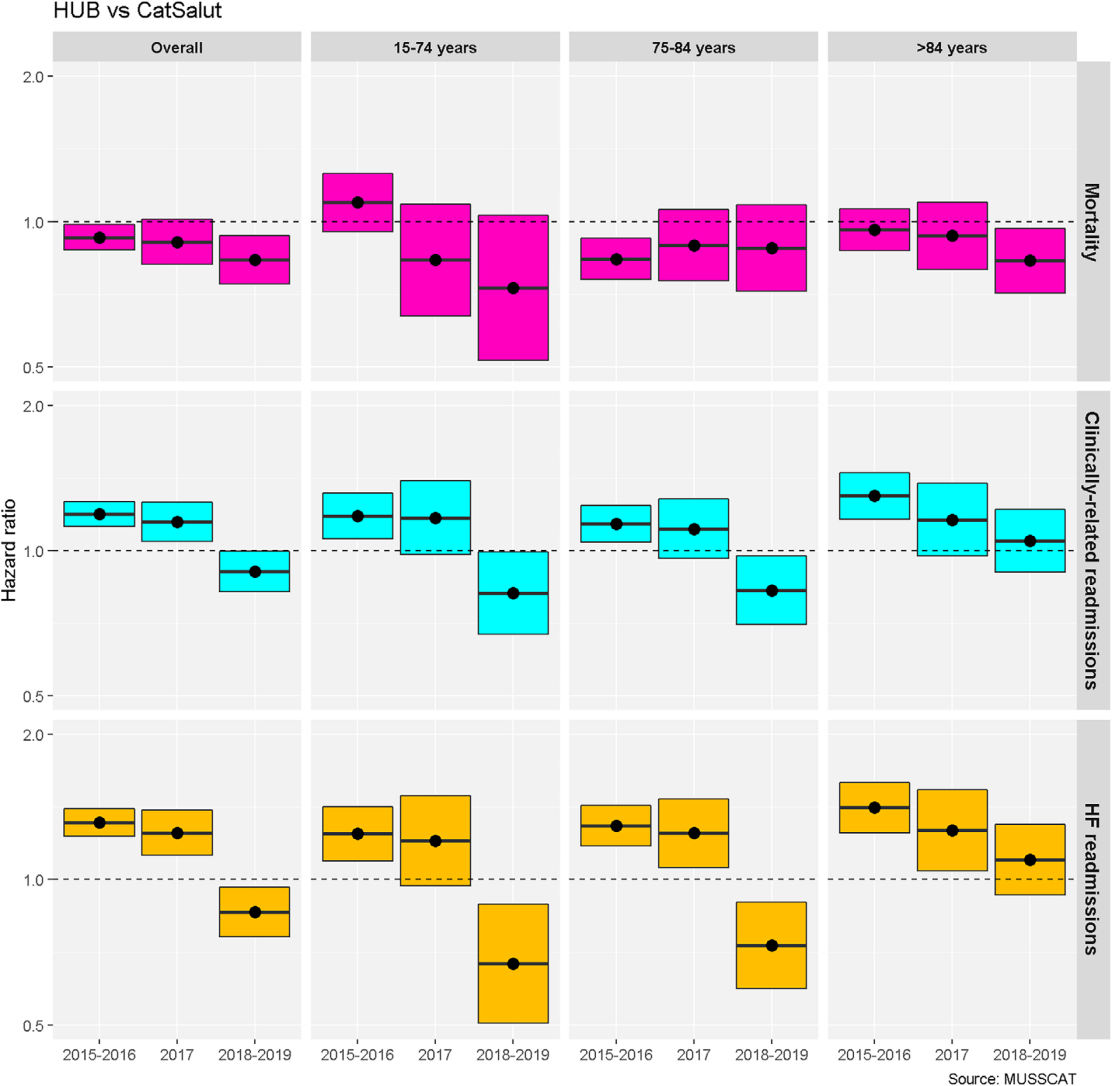
Graphical representation of heart rate and 95% confidence interval from multivariable (adjusted) Cox proportional models evaluating the impact on clinical outcomes across pre‐defined time‐periods according to healthcare setting (HUB vs. rest of the Catalan Health Service) overall and by age strata. HF, heart failure; HUB, Bellvitge University Hospital.

The implementation of the programme resulted in a significant reduction in clinically related hospitalisations, HF readmissions and all‐cause mortality in the transition and consolidation periods overall (Fig. 2); more details of the global impact have been previously published [34]. To assess the effectiveness of the HUB‐Delta programme according to age strata, several multivariate‐adjusted Cox proportional hazard analyses were performed (Table 4). All age groups experienced substantial improvement in outcomes during the consolidation period compared with the pre‐implementation period. Remarkably, a significant improvement in the risk of all‐cause mortality (hazard ratio [HR] 0.95; 95% CI 0.91–0.98; *p* = 0.003), clinically related readmission (HR 0.83; 95% CI 0.83–0.89; *p* < 0.001) and HF readmission (HR 0.85; 95% CI 0.81–0.89; *p* < 0.001) was also observed among patients in the >84 years group.

Analysis assessing the relative risk between age groups showed similar results (Table 3). In these analyses, the crude proportion of adverse events decreased across all age groups throughout the study period, as reflected by the pre‐implementation consolidation ARR values, except for all‐cause mortality in patients in the middle‐aged group. Over time, older patients tended to have worse outcomes than younger patients, except for clinically related readmissions.

**Table 3:** Proportion of occurrence of events and absolute risk reduction (ARR) according to age group strata in patients discharged alive with a primary diagnosis of HF in Bellvitge University Hospital (HUB) and Delta Primary Care Service (HUB‐Delta) healthcare area between 1 January 2015 and 31 December 2019.

	15–74 years	75–84 years	>84 years
Period	*N* (%)	*N* (%)	*N* (%)
**All‐cause mortality**			
2015–2016	428 (15%)	699 (16%)	495 (26%)
2017	183 (7.7%)	306 (16%)	235 (27%)
2018–2019	256 (5%)	362 (16%)	432 (22%)
ARR pre–post	10%	−0.1%	4%
**Clinically related readmission**			
2015–2016	428 (48%)	699 (47%)	495 (47%)
2017	183 (45%)	306 (44%)	235 (41%)
2018–2019	256 (30%)	362 (31%)	432 (33%)
ARR pre–post	18%	15%	14%
**HF readmission**			
2015–2016	428 (35%)	699 (39%)	495 (40%)
2017	183 (35%)	306 (34%)	235 (34%)
2018–2019	256 (16%)	362 (20%)	432 (27%)
ARR pre–post	20%	19%	13%

*Note*: Time is represented in pre‐defined periods. *N* corresponds to exposed patients, and % to the rate of occurrence of events. ARR represents the difference in event proportions between the pre‐implementation (2015–2016) and post‐implementation (2018–2019) periods.

Abbreviations: HF, heart failure; HUB, Bellvitge University Hospital.

### Effect of the implementation of the HF programme, comparing the HUB‐Delta area with the rest of Catalonia

To evaluate the effect of the implementation of the new HF programme in the HUB‐Delta area compared with the benchmark (rest of Catalonia) across age strata, we conducted several multivariate‐adjusted Cox proportional hazards analyses (Table 4 and Fig. 2; Table S1 and Figs. S1–S9).

**Table 4:** Multivariate (adjusted) Cox proportional hazards analyses exploring the effect on outcomes of the implementation of the primary‐care hospital integrated heart failure programme in the Bellvitge University Hospital (HUB) and Delta Primary Care Service (HUB‐Delta) healthcare area between 1 January 2015 and 31 December 2019, stratified by age.

		Outcomes in the HUB‐Delta area		
Age (years)	Period	HR	CI 95%	*p*‐value
**All‐cause mortality**				
15–74	2015–2016	Reference	–	–
	2017	0.90	0.85–0.97	0.002
	2018–2019	0.86	0.80–0.92	<0.001
75–84	2015–2016	Reference	–	–
	2017	0.91	0.87–0.95	<0.001
	2018–2019	0.90	0.86–0.94	<0.001
>84	2015–2016	Reference	–	–
	2017	0.96	0.92–1.00	0.044
	2018–2019	0.95	0.91–0.98	0.003
**Clinically related hospitalisation**				
15–74	2015–2016	Reference	–	–
	2017	0.89	0.85–0.93	<0.001
	2018–2019	0.87	0.84–0.91	<0.001
75–84	2015–2016	Reference	–	–
	2017	0.89	0.85–0.92	<0.001
	2018–2019	0.90	0.87–0.93	<0.001
>84	2015–2016	Reference	–	–
	2017	0.92	0.88–0.96	<0.001
	2018–2019	0.83	0.83–0.89	<0.001
**HF readmission**				
15–74	2015–2016	Reference	–	–
	2017	0.83	0.78–0.88	<0.001
	2018–2019	0.81	0.76–0.85	<0.001
75–84	2015–2016	Reference	–	–
	2017	0.88	0.83–0.92	<0.001
	2018–2019	0.87	0.84–0.91	<0.001
>84	2015–2016	Reference	–	–
	2017	0.90	0.86–0.95	<0.001
	2018–2019	0.85	0.81–0.89	<0.001

*Note*: Models were adjusted for sex, SES, previous hospitalisation, morbidity index (GMA: associated morbidity groups) and time since diagnosis of heart failure (HF). Time is represented in pre‐defined periods.

Abbreviations: CI, confidence interval; HR, hazard ratio.

In Catalonia, the crude proportions of adverse events also showed a downward trend across the study period (Table 5), with pre‐implementation consolidation ARR values indicating modest improvements across age groups. These changes are consistently smaller than those observed in the HUB‐Delta area (Table 4).

**Table 5:** Proportion of occurrence of events and absolute risk reduction (ARR) according to age group strata in patients discharged alive with a primary diagnosis of HF in Catalonia between 1 January 2015 and 31 December 2019.

	15–74 years	75–84 years	>84 years
Period	*N* (%)	*N* (%)	*N* (%)
**All‐cause mortality**			
2015–2016	7894 (12%)	11,772 (20%)	10,900 (31%)
2017	3990 (10%)	5250 (18%)	5341 (31%)
2018–2019	7740 (10%)	11,032 (18%)	13,635 (30%)
ARR pre–post	2.1%	1.2%	0.2%
**Clinically related readmission**			
2015–2016	7894 (42%)	11,772 (42%)	10,900 (37%)
2017	3990 (39%)	5250 (38%)	5341 (35%)
2018–2019	7740 (38%)	11,032 (38%)	13,635 (33%)
ARR pre–post	4.2%	3.7%	4.2%
**HF readmission**			
2015–2016	7894 (28%)	11,772 (30%)	10,900 (29%)
2017	3990 (24%)	5250 (26%)	5341 (27%)
2018–2019	7740 (23%)	11,032 (27%)	13,635 (25%)
ARR pre–post	5%	3.4%	4.3%

*Note*: Time is represented in pre‐defined periods. *N* corresponds to exposed patients, and % to the rate of occurrence of events. ARR represents the difference in event proportions between the pre‐implementation (2015–2016) and post‐implementation (2018–2019) periods.

Abbreviation: HF, heart failure.

Among the patients in the 15–74 years group, all‐cause mortality was lower in patients of the HUB‐Delta area compared with those in the rest of Catalonia in the consolidation period, with the highest differences observed in 2018 (HR 0.60; 95% CI 0.40–0.91; *p* = 0.015) (Table S1). All‐cause mortality tended to be lower in the middle‐aged and oldest groups during the consolidation period.

As shown in Tables 5 and 6, Fig. 2 and Figs. S4–S9, the risk of clinically related hospitalisations and HF readmissions in patients in the HUB‐Delta area was significantly lower in the consolidation period among patients in the 15–74 years age group (HR 0.77; 95% CI 0.63–0.93 and HR 0.61; 95% CI 0.46–0.80, respectively) and in the 75–84 years age group (HR 0.82, 95% CI 0.70–0.97 and HR 0.73, 95% CI 0.60–0.90, respectively) compared with the patients in these two age groups in the rest of Catalonia. Among patients in the oldest age group, the risk of HF readmissions tended to improve in the HUB‐Delta area compared with the rest of Catalonia (2015–2016 HR 1.47; 95% CI 1.30–1.66; 2018–2019 HR 1.10; 95% CI 0.93–1.30) and approached the benchmark during the consolidation period for the risk of clinically related hospitalisations (2015–2016 HR 1.35; 95% CI 1.21–1.51; 2018–2019 HR 1.05; 95% CI 0.90–1.22).

**Table 6:** Multivariate (adjusted) Cox proportional hazards analyses exploring the effect on outcomes of the implementation of the primary‐care hospital integrated heart failure programme according to age group strata in the Bellvitge University Hospital (HUB) and Delta Primary Care Service (HUB‐Delta) healthcare area versus the rest of Catalonia between 1 January 2015 and 31 December 2019.

		HUB‐Delta vs. the rest of Catalonia		
Age (year)	Period	HR	CI 95%	*p*‐value
**All‐cause mortality**				
15–74	2015–2016	1.13	0.99–1.30	0.079
	2017	0.94	0.72–1.23	0.643
	2018–2019	0.67	0.47–0.95	0.023
75–84	2015–2016	0.85	0.77–0.94	0.001
	2017	0.92	0.78–1.09	0.336
	2018–2019	0.86	0.70–1.05	0.143
>84	2015–2016	0.99	0.89–1.09	0.777
	2017	0.91	0.78–1.07	0.260
	2018–2019	0.83	0.71–0.97	0.015
**Clinically related hospitalisation**				
15–74	2015–2016	1.22	1.10–1.36	<0.001
	2017	1.25	1.05–1.49	0.014
	2018–2019	0.77	0.63–0.93	0.007
75–84	2015–2016	1.17	1.07–1.27	0.001
	2017	1.13	0.98–1.31	0.083
	2018–2019	0.82	0.70–0.97	0.019
>84	2015–2016	1.35	1.21–1.51	<0.001
	2017	1.17	0.98–1.39	0.083
	2018–2019	1.05	0.90–1.22	0.545
**HF readmission**				
15–74	2015–2016	1.31	1.15–1.49	<0.001
	2017	1.37	1.10–1.69	0.004
	2018–2019	0.61	0.46–0.80	0.001
75–84	2015–2016	1.32	1.20–1.46	<0.001
	2017	1.24	1.06–1.46	0.009
	2018–2019	0.73	0.60–0.90	0.003
>84	2015–2016	1.47	1.30–1.66	<0.001
	2017	1.25	1.03–1.52	0.022
	2018–2019	1.10	0.93–1.30	0.273

*Note*: Models were adjusted for sex, SES, previous hospitalisation, morbidity index (GMA: associated morbidity groups) and time since diagnosis of heart failure (HF). Time is represented in pre‐defined periods.

Abbreviations: CI, confidence interval; HR, hazard ratio.

## Discussion

### Summary

Our study showed that the implementation of a nurse‐led transitional care HF management programme integrating all levels of care for vulnerable patients with HF resulted in a reduction in all‐cause mortality, clinically related hospitalisations and HF readmissions across all age groups. These benefits were independent of comorbidity burden and socioeconomic status and were also observed among very old patients aged >84 years. In this group, the risk of HF readmission and clinically related hospitalisation during the consolidation period was lower than that in the middle‐aged group. We believe that this could be explained by a competing risk related to all‐cause mortality, that is, patients with a higher prevalence of comorbidities and higher frailty levels died at younger ages.

Compared with HR care in the rest of Catalonia, the programme showed significant advantages in the consolidation period across age strata: All‐cause mortality decreased significantly overall and by age group without increases in any‐cause readmissions; clinically related hospitalisations and HF readmissions were lower in the youngest and middle‐aged groups, and in those aged >84 years, risks decreased to benchmark levels.

### Limitations

Our study had some limitations that need to be addressed. First, we performed a natural experiment; therefore, data were obtained and analysed retrospectively, thus limiting the conclusions regarding causality. Nevertheless, natural experiments allow for a more realistic evaluation of the interventions, including the entire population and, more importantly, those frequently excluded, such as older patients. Second, the use of healthcare databases allowed us to capture real‐world scenarios, although it limited the availability of important clinical information, such as the aetiology of cardiomyopathy and the left ventricular ejection fraction.

### Strengths and comparison with existing literature

Our analysis is strengthened by the unprecedented use of real‐world, population‐based (i.e., whole‐cohort) data to assess interventions for older patients with HF. First, our study included all consecutive patients discharged alive after hospitalisation for HF across the entire healthcare system. Therefore, the entire population was included in the study, and typical exclusion criteria, including common age‐related comorbidities, were not present. Second, the patients were stratified by age into three different age groups: younger (15–74 years), middle‐aged (75–84 years) and older (>84 years) groups, which allowed us to explore the differences between the subgroups of older patients. Third, we evaluated outcomes in the most vulnerable phase of the disease, the post‐discharge period. Finally, the results were obtained by performing a natural experiment to observe the real impact of age on the effectiveness of implementing the new programme. These results were compared with those of the rest of Catalonia, a very similar population with universal coverage and where new different HF programmes were also implemented.

These outcomes are distinctive because of the limited information concerning treatment strategies for older patients with HF and the lack of previous studies evaluating the impact of age on the effectiveness of HF management programmes using population‐based analyses and real‐world data.

Prior studies have focused on gathering evidence for older adults with HF and their treatment strategies, generally using HF management programmes as a common base, with the same recommended structures for these programmes but with some variations [18, 19, 20]. Most of these studies were clinical trials. These are robust controlled studies in which the entire population is not well represented because of significant selection bias. Commonly, they include typical age‐related comorbidities as exclusion criteria, such as cognitive impairment or living in care facilities [21, 22, 23, 24, 25, 26, 27, 36, 37, 38].

### Implications for research and practice

Our findings are likely to be most generalisable to settings with universal coverage and well‐organised integrated care pathways that support continuity, accessibility and home‐based follow‐up. In more fragmented or non‐universal systems, differences in care coordination, resource availability and age‐related implementation barriers may limit the extent to which similar programmes can be implemented or achieve comparable outcomes.

Our programme combined components with established benefits [11, 12, 13, 14, 15, 16, 17], including proactive universal inpatient detection, coordinated interdisciplinary discharge to structured care pathways across home, primary care and hospitals, and motivational nurse‐led interventions focused on encouraging patients and caregivers to better understand and manage the disease.

We believe in the importance of evaluating the health outcomes of all the programmes implemented and using these results for the continuous improvement of programmes so that they reach the highest possible effectiveness and promote equity in healthcare delivery. We also believe in the need to represent all patients with HF, including females and older patients, in the studies and recognise the need for evidence to guide their treatment.

In conclusion, our study shows that implementing a transitional, nurse‐led HF management programme integrating all levels of care is associated with reductions in all‐cause mortality, clinically related hospitalisations and HF readmissions across all age groups. The consistent benefits observed in older adults are particularly relevant, given the limited evidence available on effective HF management strategies in this population.

Regarding future evaluations, health‐economic studies are needed to assess the cost‐effectiveness and scalability of integrated care models. Further studies should better characterise patient subgroups and ensure the inclusion of under‐represented populations, such as older or highly frail individuals, to refine patient stratification and optimise tailored care approaches.

## Author contributions

*Conceptualisation*: Blanca Torres‐Cardús, Emili Vela‐Vallespín, Cristina Enjuanes and Josep Comin‐Colet. *Methodology*: Blanca Torres‐Cardús, Emili Vela‐Vallespín, Cristina Enjuanes and Josep Comin‐Colet. *Validation*: Blanca Torres‐Cardús, Emili Vela‐Vallespín, Cristina Enjuanes and Josep Comin‐Colet. *Formal analysis*: Emili Vela‐Vallespín, David Monterde, Gerard Carot‐Sans, Jordi Piera‐Jiménez, Júlia Folguera and Josep Comin‐Colet. *Investigation and resources*: Blanca Torres‐Cardús, Cristina Enjuanes and Josep Comin‐Colet. *Data curation*: Emili Vela‐Vallespín, David Monterde, Gerard Carot‐Sans, Jordi Piera‐Jiménez and Júlia Folguera. *Interpretation of data*: Blanca Torres‐Cardús, Lídia Alcoberro Torres, Raúl Ramos‐Polo, Encarnación Hidalgo‐Quirós, Núria José‐Bazán, Sergi Yun Viladomat, Pedro Moliner, Santiago Jiménez‐Marrero, Alberto Garay Melero, Alexandra Pons Riverola, Lídia Alcoberro Torres, Herminio Morillas, Betlem Salvador‐González, Santiago Jiménez‐Marrero, Mireia Andrés, Cristina Enjuanes and Josep Comin‐Colet. *Writing—original draft preparation*: Blanca Torres‐Cardús, Cristina Enjuanes and Josep Comin‐Colet. *Writing—review and editing*: Blanca Torres‐Cardús, Cristina Enjuanes, Emili Vela‐Vallespín, David Monterde, Gerard Carot‐Sans, Jordi Piera‐Jiménez, Júlia Folguera, Lídia Alcoberro Torres, Raúl Ramos‐Polo, Encarnación Hidalgo‐Quirós, Núria José‐Bazán, E.C., Sergi Yun Viladomat, Pedro Moliner, Santiago Jiménez‐Marrero, Alberto Garay Melero, Alexandra Pons Riverola, Lídia Alcoberro Torres, Herminio Morillas, Betlem Salvador‐González, Santiago Jiménez‐Marrero, Mireia Andrés and Josep Comin‐Colet. *Supervision*: Cristina Enjuanes and Josep Comin‐Colet. Project administration: Josep Comin‐Colet. *Funding acquisition*: Josep Comin‐Colet. All authors agree to be accountable for all aspects of the work and ensure that questions related to the accuracy or integrity of any part of the work are appropriately investigated and resolved.

## Conflict of interest statement

The authors declare no conflicts of interest.

## Funding information

Department of Health of the Government of Catalonia through the ‘Pla Estratègic de Recerca i Innovació en Salut (PERIS 2023)’ grant number SLT028/23/000018.

## Supporting information



Supplementary materials are available in the document *Supplementary Appendix for Complementary Data and Materials*.
**Table S1**. Multivariate (adjusted) Cox proportional hazards analyses exploring the effect on outcomes of the implementation of the primary‐care hospital integrated heart failure programme according to age group strata in the HUB‐Delta healthcare area between 1 January 2015 and 31 December 2019. Models were adjusted for sex, SES, previous hospitalisation, morbidity index (GMA: associated morbidity groups) and time since diagnosis of HF.
**Figure S1**. Survival curves were estimated on the basis of multivariate (adjusted) Cox models evaluating the impact on adjusted probability of all‐cause mortality according to healthcare setting (HUB‐Delta vs. rest of Catalonia) across predefined periods: 2015–2016 (Panel A), 2017 (Panel B) and 2018–2019 (Panel C) in the age group 15–74.
**Figure S2**. Survival curves were estimated on the basis of multivariate (adjusted) Cox models evaluating the impact on adjusted probability of all‐cause mortality according to healthcare setting (HUB‐Delta vs. rest of Catalonia) across predefined periods: 2015–2016 (Panel A), 2017 (Panel B) and 2018–2019 (Panel C) in the age group 75–84.
**Figure S3**. Survival curves were estimated on the basis of multivariate (adjusted) Cox models evaluating the impact on adjusted probability of all‐cause mortality according to healthcare setting (HUB‐Delta vs. rest of Catalonia) across predefined periods: 2015–2016 (Panel A), 2017 (Panel B) and 2018–2019 (Panel C) in the age group >84.
**Figure S4**. Survival curves were estimated on the basis of multivariate (adjusted) Cox models evaluating the impact on adjusted probability of clinically related readmission according to healthcare setting (HUB‐Delta vs. rest of Catalonia) across predefined periods: 2015–2016 (Panel A), 2017 (Panel B) and 2018–2019 (Panel C) in the age group 15–74.
**Figure S5**. Survival curves were estimated on the basis of multivariate (adjusted) Cox models evaluating the impact on adjusted probability of clinically related readmission according to healthcare setting (HUB‐Delta vs. rest of Catalonia) across predefined periods: 2015–2016 (Panel A), 2017 (Panel B) and 2018–2019 (Panel C) in the age group 75–84.
**Figure S6**. Survival curves were estimated on the basis of multivariate (adjusted) Cox models evaluating the impact on adjusted probability of clinically related readmission according to healthcare setting (HUB‐Delta vs. rest of Catalonia) across predefined periods: 2015–2016 (Panel A), 2017 (Panel B) and 2018–2019 (Panel C) in the age group >84.
**Figure S7**. Survival curves were estimated on the basis of multivariate (adjusted) Cox models evaluating the impact on adjusted probability of HF readmission according to healthcare setting (HUB‐Delta vs. rest of Catalonia) across predefined periods: 2015–2016 (Panel A), 2017 (Panel B) and 2018–2019 (Panel C) in the age group 15–74.
**Figure S8**. Survival curves were estimated on the basis of multivariate (adjusted) Cox models evaluating the impact on adjusted probability of HF readmission according to healthcare setting (HUB‐Delta vs. rest of Catalonia) across predefined periods: 2015–2016 (Panel A), 2017 (Panel B) and 2018–2019 (Panel C) in the age group 75–84.
**Figure S9**. Survival curves were estimated on the basis of multivariate (adjusted) Cox models evaluating the impact on adjusted probability of HF readmission according to healthcare setting (HUB‐Delta vs. rest of Catalonia) across predefined periods: 2015–2016 (Panel A), 2017 (Panel B) and 2018–2019 (Panel C) in the age group >84.

## Data Availability

The data that support the findings of this study are available on request from the corresponding author. The data are not publicly available due to privacy or ethical restrictions.
