# Supplementary material for: Age‐stratified effectiveness of a nurse‐led heart failure programme: A population‐based study
Source: J Intern Med. 2026 Jul 12;300(3):345–59. doi: 10.1111/joim.70136 (PMC13429005; doi:10.1111/joim.70136)

**SUPPLEMENTARY APPENDIX FOR COMPLEMENTARY DATA AND MATERIALS**

**SECTION A: STUDY CONTEXT AND COMPONENTS AND IMPLEMENTATION OF THE NEW INTEGRATED HEART FAILURE PROGRAMME**

This study was conducted in Catalonia. Catalonia is located in the Northeast of Spain. As in most European regions, in Catalonia, residents (N = 7,816,419 in 2019) are granted universal public healthcare coverage by law.

In Catalonia, healthcare is delivered by the Catalan Health Service (CHS). The Catalan territory is divided into seven health regions, delimited on the basis of geographical, socio-economic, and demographic factors. They have an adequate endowment of health resources for primary and specialised care including hospital care to meet the needs of the population. Each region is ordered into health sectors, and these sectors are in turn, composed of integrated healthcare areas where coordination of care between hospital and primary care is contractually warranted.

In recent years, specialised disease management programmes, improved transitional care, and enhanced long-term care, among other interventions, have been developed in Catalonia by the CHS to promote quality-of-care improvement for patients with chronic conditions, particularly chronic HF [12,16,21].

Since 2017, a new programme to improve quality of care of patients with chronic HF has been developed and implemented in the Bellvitge University Hospital-Delta Primary Care Service (HUB-Delta) integrated healthcare area, located in the South Metropolitan Sector of the Barcelona Health Region. The HUB-Delta integrated healthcare area provides multilevel and multi-provider healthcare coordination for a population of 209,255 (2019) in the city of Prat del Llobregat and two urban districts of the city of Hospitalet de Llobregat (south and centre Hospitalet). The healthcare network for patients with HF constructed in the HUB-Delta area integrates healthcare institutions belonging to the Catalan Health Institute that include Bellvitge University Hospital and 10 primary care centres of the Delta Primary Care Service along with other providers (health and social care, rehabilitation services, social services).

The HUB-Delta HF programme was designed as a nurse-led multidisciplinary, transitional care, and nurse-led programme. Details of the model implemented have been previously published [34]. In its design, an attempt was made to develop the conceptual framework provided by the Chronic Care Model, which includes all components of care and interventions that have shown benefits in HF patients [13-14,16,28-29]. In brief, these include: 1) encouraging patient empowerment through promotion of self-management and self-efficacy, 2) promoting proactive planned structured care interventions instead of reactive care, 3) interventions based on advanced practice nurses in both hospital and primary care, 4) a multidisciplinary team approach, 5) promotion of flexible healthcare services providing open access to patients when needed, 6) prioritisation of eHealth-based care (telemedicine), 7) implementation of the use of electronic tools and strategies to support decision-making to specialised nurses, community nurses, and family physicians, 8) enabling the use of shared electronic medical records and information systems to, first, improve communication and coordination of care among healthcare professionals and, second, to provide information to clinicians and managers on outcomes using dedicated key performance indicators (KPI) obtained from the information systems.

This model was successfully implemented previously by our group in a different healthcare area [12]. In the current implementation, the model has been updated and improved in several aspects including: 1) a more refined universal detection of patients in the acute phase, 2) enhanced discharge coordination, 3) improved early post-discharge contact, 4) extension of structured follow up pathways of patients in all care settings including hospital-based HF clinic, primary care offices and home based care, 5) shared electronic care plans between hospital and primary care, 6) motivational interviewing-based nurse interventions and 7) robust KPI monitoring of the implementation.

**SECTION B: DATA SOURCES AND DATA QUALITY CONTROL**

Since 2011, the Health Department of the Government of Catalonia uses an automated administrative healthcare database (the Catalan Health Surveillance System [CHSS]), which periodically collects detailed individual-level information on demographics and socioeconomic characteristics, as well as exhaustive health-related and medical resource use information generated by the interactions between Catalan residents and the public healthcare system. This longitudinal, quality-controlled, updated information system allows the conduct of epidemiological analyses, evaluations of healthcare interventions and programmes, and public analysis and benchmarking of health indicators across healthcare areas, among other assessments. Further details on the characteristics of the CHSS database have been reported elsewhere by our group [1,3,30-31,34].

Specifically for healthcare-related data, the database integrates information from a number of sources, including the Minimum Basic Dataset for Healthcare Units registry (which includes hospitalisations and use of primary care, emergency department, outpatient hospital clinics, mental health services and skilled nursing facility services), information on pharmacy prescription, dialysis, ambulatory rehabilitation, home-based respiratory therapies, non-urgent medical transport and billing records among other information. Medical conditions are coded using the International Classification of Diseases, 9th Revision, Clinical Modification (ICD-9-CM) coding system. Vital status of the citizens included in the database is updated using information from the Spanish National Statistics Institute. These registries are subject to automated data validation, and external audits are conducted periodically to ensure data quality.

**SECTION C: STUDY DESIGN, STUDY POPULATION, CODING CRITERIA AND OBJECTIVES**

The design of this study has been previously published [34]. In summary, to evaluate the influence of age on the effectiveness of a multilevel, multidisciplinary, transitional care programme for the management of patients with HF we followed three critical steps.

In the first step, we designed and implemented a comprehensive HF programme in the HUB-Delta integrated healthcare area between November 2016 and December 2019. We consider three distinct implementation periods: pre-implementation period (2015-2016), transition period (2017) and consolidation period (2018- 2019).

As a second step, we designed a pragmatic, population-based evaluation of the implementation of the programme conducting a natural experiment. For the purposes of this study, we included all individuals consecutively admitted to hospital with at least one ICD-9-CM code for HF as the primary diagnosis in Catalonia between 1 January 2015 and 31 December 2019. ICD-9-CM codes used for hospital admission due to HF were: 398.91, 402.x1, 404.x1, 404.x3, 428.0, 428.1, 428.2x, 428.x3, and 428.x4. Only patients discharged alive were considered for the analysis. Age, general clinical characteristics, demographic information, information on comorbidities and previous medical resource use were obtained at baseline in all cases. Clinical outcomes were measured and analysed for all patients between 1 January 2015 and 31 December 2019 across pre-determined age groups: a young group aged from 15 to 74 years, a middle-age group aged from 75 to 84 years, and an old group, of those aged above 84 years. For the index admission at each time-period or year of analysis and successive clinically-related and HF readmissions, we considered only unplanned acute admissions of more than 24 hours duration.

The third step of the current project involved the evaluation of the effectiveness of the HUB-Delta HF programme stratified according to age across the studied time periods.

We measured effectiveness of the implementation of the programme across age strata at two levels: first, comparing the outcomes of patients exposed to the HUB-Delta HF programme between periods of implementation taking the year 2015 (pre-implementation) and the period 2015-2016 (pre-implementation period) as the reference year and period (intragroup comparison) and, second, comparing outcomes between patients of the HUB-Delta area with patients of the rest of the population covered by the CHSS at each predefined period (between groups comparison).

The primary outcome variable of the present analysis was the time to the first clinically-related readmission at each specific age group. Secondary outcome variables were time to the first admission for HF and time to death at each specific age group.

The outcomes variables were evaluated globally for the evaluation of the newly implemented HF programme and stratified according to age for the evaluation of the influence of age on the effectiveness of a comprehensive HF programme. The complete coding criteria for the present study are presented in Supplementary Table S0 (see below).

**SECTION D: ASSESSMENT OF SOCIOECONOMIC STATUS, COVARIATES, QUALITY-OF-CARE INDICATORS, AND STUDY ENDPOINTS**

**Assessment of individual socioeconomic status**

Methodology regarding the assessment of individual socioeconomic status has been previously reported by our group [30-31,34]. Briefly, in the CHSS database, information on individual annual income (classified as <18,000€, 18,000–100,000€, >100,000€) is recorded and updated on a yearly basis, as well as information on receipt of welfare economic support by the Government. For the purposes of our study, these variables were assessed for all patients at the time the index admission occurred. We defined four individual income categories: an annual income >100,000€ was considered “high” income, 18,000–100,000€ was considered “medium” income, and <18,000€ was considered “low” income. These three categories included both active workers as well as retired individuals receiving a retirement pension. Finally, individuals who received welfare support by the Government were considered to have “very low income”. This categorisation mirrors the one used in Catalonia for pharmaceutical co-payment purposes. For the present analysis, we grouped patients with medium and high income in a single category. Information on education level was not available in the database.

**Assessment of covariates and comorbidities**

Information on other relevant covariates including sex and comorbidities available at the time the index admission occurred was used for all patients. Specifically for comorbidities, we used the “adjusted morbidity groups” (GMA [Catalan acronym for “Grups de Morbiditat Ajustats”]) comorbidity classification system [32,35]. The GMA system has been developed specifically for the CHS and includes 31 mutually exclusive categories of morbidity and complexity (social features not included) and can be expressed as a single GMA index. Importantly, the GMA system has shown to outperform Charlson Comorbidity Index in the Catalan population in terms of their ability to predict incident urgent hospitalisations. For the present study, based on the distribution of GMAs within the HF study population, four GMA risk strata were defined: low, intermediate, high and very high.

For covariate specification, previous hospitalisation (operationalised as a count variable) was defined as any all-cause hospital admission recorded in the CHSS prior to the index HF hospitalisation and parameterised as a count variable (total number of prior admissions). The look-back window encompassed the full historical utilisation period available for each patient within the CHSS.

**Assessment of quality-of-care indicators**

The measure of the quality, complexity and intensity of the new HF programme has been previously assessed and published by our group [34].

**Ascertainment of study endpoints**

Urgent clinically-related hospitalisation (i.e., unplanned hospitalisation, as opposed to planned hospitalisations, such as those for diagnostic or surgical procedures) was the primary endpoint of our study. HF re-hospitalisations and all-cause mortality were the secondary endpoints of the study.

Clinically-related hospital readmissions were defined using the Chronic Condition Indicator criteria for the ICD-9-CM of the Agency for Healthcare Research and Quality [33].

According to this, clinically-related readmissions can be catalogued as:

1) Recurrences: Discharges with a primary diagnosis of circulatory or heart disease (07-Diseases of the circulatory System, according to CCI), or a primary diagnosis of acute respiratory failure and principal secondary diagnosis of heart failure with no external cause.

2) Admissions due to pre-existing chronic conditions (non-circulatory) and without external cause.

3) Admissions due to complications of care (iatrogenic/procedure-related). The list of complications is listed in Supplementary Table S0 (Iatrogenic complications).

HF readmissions were defined using the same coding criteria used for the index HF admission and are also listed in the Supplementary Table S0.

**SECTION E: STATISTICAL ASPECTS OF THE STUDY**

The baseline characteristics of the study population were described overall and by several strata including healthcare area (CHS vs. HUB-Delta), periods of study and age groups. Categorical variables were reported using number and proportion, and continuous variables using mean and standard deviation. Characteristics were compared across the different strata using chi-squared, Student’s t-test, One-way ANOVA or non-parametric tests, as appropriate. For descriptive analyses, absolute risk reduction (ARR) was calculated as the absolute difference in crude event proportions between the pre‑implementation (2015–2016) and consolidation (2018–2019) periods in both regions

First, multivariate adjusted Cox proportional hazards models analysing the risk of occurrence of clinically-related readmission, HF readmission, and all-cause mortality according to age group strata were conducted in patients discharged alive with a primary diagnosis of HF in Catalonia between 1 January 2015 and 31 December 2019. All models were adjusted for sex, age, individual socioeconomic status, previous hospitalisation, comorbidities using the GMA index and time since diagnosis of HF.

Following these, the proportion of the occurrence of clinical endpoints according to age group strata after discharge across the predefined time-periods was explored using multivariate Cox proportional hazards models in patients discharged alive with a primary diagnosis of HF in Catalonia and the HUB-Delta area between 1 January 2015 and 31 December 2019.

Furthermore, multivariate Cox proportional hazards models were used to assess the effect on clinical outcomes of the implementation of the hospital-primary care integrated HF programme in the HUB-Delta healthcare area stratified by age. Primary and secondary endpoints were analysed across years and pre-defined time-periods according to healthcare setting in models adjusted for age, sex, individual socioeconomic status, previous hospitalisation, comorbidities using GMA index and time since diagnosis of HF.

In addition, to explore the effect of the programme according to age group strata, several multivariable (adjusted) Cox proportional models evaluating the impact on clinical outcomes across pre-defined time-periods according to healthcare setting (HUB vs. rest of CHS) and stratified by age group strata were conducted. The effect of the interaction between age groups and healthcare area (HUB-Delta vs. rest of CHS) was further explored in similar models.

For this analysis, the adjusted probabilities of experiencing any of the clinical events studied here are graphically represented, according to the period, and stratified by age group strata based on Cox proportional hazards models.

All statistical tests and confidence intervals (CI) were constructed with a type I error alpha level of 5%, with no adjustments for multiplicity. P values below 0.05 were considered statistically significant. All analyses were performed using R software (version 4.0.2; R Foundation for Statistical Computing, Vienna, Austria).

**SECTION F. SUPPLEMENTARY TABLES**

| **Supplementary Table S0.** ICD-9-CM codes used to define each of the conditions evaluated in the study.  **Previous MI**   \| **Code** \| **Description** \| \| --- \| --- \| \| 410.xx \| Acute myocardial infarction \| \| 412 \| Old myocardial infarction \|   **Atrial fibrillation**   \| **Code** \| **Description** \| \| --- \| --- \| \| 427.31 \| Atrial fibrillation \|   **Peripheral Vascular Disease**   \| **Code** \| **Description** \| \| --- \| --- \| \| 093.0 \| Aneurysm of aorta specified as syphilitic \| \| 437.3 \| Cerebral aneurysm non-ruptured \| \| 440.x \| Atherosclerosis \| \| 441.x \| Aortic aneurysm and dissection \| \| 443.1 \| Thromboangiitis obliterans (Buerger's disease) \| \| 443.2x \| Other peripheral vascular disease \| \| 443.8x \| Other specified peripheral vascular diseases \| \| 443.9 \| Peripheral vascular disease unspecified \| \| 447.1 \| Stricture of artery \| \| 557.1 \| Chronic vascular insufficiency of intestine \| \| 557.9 \| Unspecified vascular insufficiency of intestine \| \| V43.4 \| Blood vessel replaced by other means \|   **Hypertension**   \| **Code** \| **Description** \| \| --- \| --- \| \| 401.xx \| Essential hypertension \| \| 402.xx \| Hypertensive heart disease \| \| 403.xx \| Hypertensive renal disease \| \| 404.xx \| Hypertensive heart and renal disease \| \| 405.xx \| Secondary hypertension \|   **Obesity**   \| **Code** \| **Description** \| \| --- \| --- \| \| 278.00 \| Obesity, unspecified \| \| 278.01 \| Morbid obesity \| \| V85.3x \| Body Mass Index between 30-39, adult \| \| V85.4x \| Body Mass Index 40 and over, adult \|   **Smoking habit**   \| **Code** \| **Description** \| \| --- \| --- \| \| 305.1 \| Tobacco use disorder \| \| 649.0x \| Tobacco use disorder complicating pregnancy, childbirth, or the puerperium \| \| 989.84 \| Toxic effect of other substances: Tobacco \| \| V15.82 \| History of tobacco use \|   **Hyperlipidaemia**   \| **Code** \| **Description** \| \| --- \| --- \| \| 272.0x \| Pure hypercholesterolemia \| \| 272.1x \| Pure hyperglyceridaemia \| \| 272.2x \| Hyperlipidaemia, mixed \| \| 272.3x \| Hyperchylomicronaemia \| \| 272.4x \| Other and unspecified hyperlipidaemia \|   **Diabetes Mellitus**   \| **Code** \| **Description** \| \| --- \| --- \| \| 250.xx \| Diabetes mellitus \|   **CKD (chronic kidney disease)**   \| **Code** \| **Description** \| \| --- \| --- \| \| 403.01 \| Hypertensive chronic kidney disease, malignant, with chronic kidney disease stage v or end stage renal disease \| \| 403.11 \| Hypertensive chronic kidney disease, benign, with chronic kidney disease stage v or end stage renal disease \| \| 403.91 \| Hypertensive chronic kidney disease, unspecified, with chronic kidney disease stage v or end stage renal disease \| \| 404.02 \| Hypertensive heart and chronic kidney disease, malignant, without heart failure and with chronic kidney disease stage v or end stage renal disease \| \| 404.03 \| Hypertensive heart and chronic kidney disease, malignant, with heart failure and with chronic kidney disease stage v or end stage renal disease \| \| 404.12 \| Hypertensive heart and chronic kidney disease, benign, without heart failure and with chronic kidney disease stage v or end stage renal disease \| \| 404.13 \| Hypertensive heart and chronic kidney disease, benign, with heart failure and chronic kidney disease stage v or end stage renal disease \| \| 404.92 \| Hypertensive heart and chronic kidney disease, unspecified, without heart failure and with chronic kidney disease stage v or end stage renal disease \| \| 404.93 \| Hypertensive heart and chronic kidney disease, unspecified, with heart failure and chronic kidney disease stage v or end stage renal disease \| \| 582.x \| Chronic glomerulonephritis \| \| 583.0x \| Nephritis and nephropathy not specified as acute or chronic with lesion of proliferative glomerulonephritis \| \| 583.1x \| Nephritis and nephropathy not specified as acute or chronic with lesion of membranous glomerulonephritis \| \| 583.2x \| Nephritis and nephropathy not specified as acute or chronic with lesion of membranoproliferative glomerulonephritis. \| \| 583.4x \| Nephritis and nephropathy not specified as acute or chronic with lesion of rapidly progressive glomerulonephritis \| \| 583.6x \| Nephritis and nephropathy not specified as acute or chronic with lesion of renal cortical necrosis \| \| 583.7x \| Nephritis and nephropathy not specified as acute or chronic with lesion of renal medullary necrosis \| \| 585.x \| Chronic renal failure \| \| 586.x \| Renal failure unspecified \| \| 588.0 \| Renal osteodystrophy \| \| V42.0 \| Kidney replaced by transplant \| \| V45.1x \| Postsurgical renal dialysis status \| \| V56.x \| Encounter for dialysis and dialysis catheter care \|   **Anaemia**   \| **Code** \| **Description** \| \| --- \| --- \| \| 280.x \| Iron deficiency anaemias \| \| 281.x \| Other deficiency anaemias \| \| 282.x \| Hereditary haemolytic anaemias \| \| 283.x \| Acquired haemolytic anaemias \| \| 284.x \| Aplastic anaemia \| \| 285.x \| Other and unspecified anaemias \|   **COPD (Chronic obstructive pulmonary disease)**   \| **Code** \| **Description** \| \| --- \| --- \| \| 491.0 \| Chronic bronchitis \| \| 491.2x \| Obstructive chronic bronchitis \| \| 491.8 \| Other chronic bronchitis \| \| 491.9 \| Unspecified chronic bronchitis \| \| 492.x \| Emphysema \| \| 494.x \| Bronchiectasis \| \| 496 \| Chronic airway obstruction, not elsewhere classified \|   **Cancer**   \| **Code** \| **Description** \| \| --- \| --- \| \| 140.x \| Malignant neoplasm of lip \| \| 141.x \| Malignant neoplasm of tongue \| \| 142.x \| Malignant neoplasm of major salivary glands \| \| 143.x \| Malignant neoplasm of gum \| \| 144.x \| Malignant neoplasm of floor of mouth \| \| 145.x \| Malignant neoplasm of other and unspecified parts of mouth \| \| 146.x \| Malignant neoplasm of oropharynx \| \| 147.x \| Malignant neoplasm of nasopharynx \| \| 148.x \| Malignant neoplasm of hypopharynx \| \| 149.x \| Malignant neoplasm of other and ill-defined sites within the lip oral cavity and pharynx \| \| 150.x \| Malignant neoplasm of esophagus \| \| 151.x \| Malignant neoplasm of stomach \| \| 152.x \| Malignant neoplasm of small intestine including duodenum \| \| 153.x \| Malignant neoplasm of colon \| \| 154.x \| Malignant neoplasm of rectum rectosigmoid junction and anus \| \| 155.x \| Malignant neoplasm of liver and intrahepatic bile ducts \| \| 156.x \| Malignant neoplasm of gallbladder and extrahepatic bile ducts \| \| 157.x \| Malignant neoplasm of pancreas \| \| 158.x \| Malignant neoplasm of retroperitoneum and peritoneum \| \| 159.x \| Malignant neoplasm of other and ill-defined sites within the digestive organs and peritoneum \| \| 160.x \| Malignant neoplasm of nasal cavities middle ear and accessory sinuses \| \| 161.x \| Malignant neoplasm of larynx \| \| 162.x \| Malignant neoplasm of trachea bronchus and lung \| \| 163.x \| Malignant neoplasm of pleura \| \| 164.x \| Malignant neoplasm of thymus heart and mediastinum \| \| 165.x \| Malignant neoplasm of other and ill-defined sites within the respiratory system and intrathoracic organs \| \| 170.x \| Malignant neoplasm of bone and articular cartilage \| \| 171.x \| Malignant neoplasm of connective and other soft tissue \| \| 172.x \| Malignant melanoma of skin \| \| 173.x \| Other malignant neoplasms of skin \| \| 174.x \| Malignant neoplasm of female breast \| \| 175.x \| Malignant neoplasm of female breast \| \| 176.x \| Kaposi’s Sarcoma \| \| 179.x \| Malignant neoplasm of uterus-part unspecified \| \| 180.x \| Malignant neoplasm of cervix uteri \| \| 181.x \| Malignant neoplasm of placenta \| \| 182.x \| Malignant neoplasm of body of uterus \| \| 183.x \| Malignant neoplasm of ovary and other uterine adnexa \| \| 184.x \| Malignant neoplasm of other and unspecified female genital organs. \| \| 185.x \| Malignant neoplasm of prostate \| \| 186.x \| Malignant neoplasm of testis \| \| 187.x \| Malignant neoplasm of penis and other male genital organs \| \| 188.x \| Malignant neoplasm of bladder \| \| 189.x \| Malignant neoplasm of kidney and other and unspecified urinary organs \| \| 190.x \| Malignant neoplasm of eye \| \| 191.x \| Malignant neoplasm of brain \| \| 192.x \| Malignant neoplasm of other and unspecified parts of nervous system \| \| 193.x \| Malignant neoplasm of thyroid gland \| \| 194.x \| Malignant neoplasm of other endocrine glands and related structures \| \| 195.x \| Malignant neoplasm of other and ill-defined sites \| \| 196.x \| Secondary and unspecified malignant neoplasm of lymph nodes \| \| 197.x \| Secondary malignant neoplasm of respiratory and digestive systems \| \| 198.x \| Secondary malignant neoplasm of other specified sites \| \| 199.x \| Malignant neoplasm without specification of site \| \| 200.x \| Lymphosarcoma and reticulosarcoma \| \| 201.x \| Hodgkin's disease \| \| 202.x \| Other malignant neoplasms of lymphoid and histiocytic tissue \| \| 203.x \| Multiple myeloma and immunoproliferative neoplasms \| \| 204.x \| Lymphoid leukaemia \| \| 205.x \| Myeloid leukaemia \| \| 206.x \| Monocytic leukaemia \| \| 207.x \| Other specified leukaemia’s \| \| 208.x \| Leukaemia of unspecified cell type \| \| 209.1x \| Malignant carcinoid tumours of the appendix large intestine and rectum \| \| 209.2x \| Malignant carcinoid tumour of unknown primary site \| \| 209.3x \| Malignant poorly differentiated neuroendocrine carcinoma any site \| \| 209.7x \| Secondary neuroendocrine tumours \| \| 230.x \| Carcinoma in situ of digestive organs \| \| 231.x \| Carcinoma in situ of respiratory system \| \| 232.x \| Carcinoma in situ of skin \| \| 233.x \| Carcinoma in situ of breast and genitourinary system \| \| 234.x \| Carcinoma in situ of other and unspecified sites \|   **Osteoarthritis**   \| **Code** \| **Description** \| \| --- \| --- \| \| 712.x \| Crystal arthropathies \| \| 713.x \| Arthropathy associated with other disorders classified elsewhere \| \| 714.x \| Rheumatoid arthritis and other inflammatory polyarthropathies \| \| 716.x \| Other and unspecified arthropathies \| \| 720.0x \| Ankylosing spondylitis \| \| 730.x \| Osteomyelitis periostitis and other infections involving bone \|   **Cognitive impairment**   \| **Code** \| **Description** \| \| --- \| --- \| \| 317 \| Mild intellectual disabilities \| \| 318.x \| Other specified mental retardation \| \| 319 \| Unspecified intellectual disabilities \|   **Cirrhosis**   \| **Code** \| **Description** \| \| --- \| --- \| \| 571.2 \| Alcoholic cirrhosis of liver \| \| 571.5 \| Cirrhosis of liver without alcohol \|   **Major mental health disorder**   \| **Code** \| **Description** \| \| --- \| --- \| \| 295.0x \| Simple type schizophrenia \| \| 295.1x \| Disorganised type schizophrenia \| \| 295.2x \| Catatonic type schizophrenia \| \| 295.3x \| Paranoid type schizophrenia \| \| 295.5x \| Latent schizophrenia \| \| 295.6x \| Residual schizophrenia \| \| 295.7x \| Schizo-affective type schizophrenia \| \| 295.8x \| Other specified types of schizophrenia \| \| 295.9x \| Unspecified schizophrenia \| \| 301.22 \| Schizotypal personality disorder \| \| 297.1 \| Delusional disorder \| \| 297.3 \| Shared psychotic disorder \| \| 296.4x \| Bipolar I disorder, most recent episode (or current) manic \| \| 296.5x \| Bipolar I disorder, most recent episode (or current) depressed \| \| 296.6x \| Bipolar I disorder, most recent episode (or current) mixed \| \| 296.7 \| Bipolar I disorder, most recent episode (or current) unspecified \| \| 296.8x \| Other and unspecified bipolar disorders \| \| 296.3x \| Major depressive disorder recurrent episode \| \| 297.9 \| Unspecified paranoid state \| \| 298.9 \| Unspecified psychosis \| \| 300.21 \| Agoraphobia with panic disorder \| \| 300.3 \| Obsessive-compulsive disorders \| \| 303.x \| Alcohol dependence syndrome \| \| 304.2x \| Cocaine dependence \| \| 304.0x \| Opioid type dependence \| \| 304.7x \| Combinations of opioid type drug with any other drug dependence \| \| 299.x \| Pervasive developmental disorders \| \| 307.1 \| Anorexia nervosa \| \| 307.51 \| Bulimia nervosa \|   **Alcohol abuse**   \| **Code** \| **Description** \| \| --- \| --- \| \| 303.x \| Alcohol dependence syndrome \| \| 305.0x \| Nondependent alcohol abuse \|   **Opioid abuse**   \| **Code** \| **Description** \| \| --- \| --- \| \| 304.0x \| Opioid type dependence \| \| 304.7x \| Combinations of opioid type drug with any other drug dependence \| \| 305.5x \| Nondependent opioid abuse \|   **Cocaine abuse**   \| **Code** \| **Description** \| \| --- \| --- \| \| 304.2x \| Cocaine dependence \| \| 305.6x \| Nondependent cocaine abuse \|   **Heart failure**   \| **Code** \| **Description** \| \| --- \| --- \| \| 398.91 \| Rheumatic heart failure (congestive) \| \| 402.01 \| Malignant hypertensive heart disease with heart failure \| \| 402.11 \| Benign hypertensive heart disease with heart failure \| \| 402.91 \| Unspecified hypertensive heart disease with heart failure \| \| 404.01 \| Hypertensive heart and chronic kidney disease, malignant, with heart failure and with chronic kidney disease stage I through stage IV, or unspecified \| \| 404.03 \| Hypertensive heart and chronic kidney disease, malignant, with heart failure and with chronic kidney disease stage V or end stage renal disease \| \| 404.11 \| Hypertensive heart and chronic kidney disease, benign, with heart failure and with chronic kidney disease stage I through stage IV, or unspecified \| \| 404.13 \| Hypertensive heart and chronic kidney disease, benign, with heart failure and chronic kidney disease stage V or end stage renal disease \| \| 404.91 \| Hypertensive heart and chronic kidney disease, unspecified, with heart failure and with chronic kidney disease stage I through stage IV, or unspecified \| \| 404.93 \| Hypertensive heart and chronic kidney disease, unspecified, with heart failure and chronic kidney disease stage V or end stage renal disease \| \| 428.x \| Heart failure \|   **Iatrogenic complications**   \| **Code** \| **Description** \| \| --- \| --- \| \| 244.0 \| Postsurgical hypothyroidism \| \| 244.1 \| Other postablative hypothyroidism \| \| 244.2 \| Iodine hypothyroidism \| \| 244.3 \| Other iatrogenic hypothyroidism \| \| 245.4 \| Iatrogenic thyroiditis \| \| 251.0 \| Hypoglycaemic coma \| \| 251.3 \| Postsurgical hypoinsulinemia \| \| 253.7 \| Iatrogenic pituitary disorders \| \| 276.61 \| Transfusion associated circulatory overload \| \| 277.83 \| Iatrogenic carnitine deficiency \| \| 277.88 \| Tumour lysis syndrome \| \| 285.3 \| Antineoplastic chemotherapy induced anaemia \| \| 287.41 \| Posttransfusion purpura \| \| 338.12 \| Acute post-thoracotomy pain \| \| 338.18 \| Other acute postoperative pain \| \| 338.22 \| Chronic post-thoracotomy pain \| \| 338.28 \| Other chronic postoperative pain \| \| 349.0 \| Reaction to spinal or lumbar puncture \| \| 349.1 \| Nervous system complications from surgically implanted device \| \| 349.31 \| Accidental puncture or laceration of dura during a procedure \| \| 415.11 \| Iatrogenic pulmonary embolism and infarction \| \| 429.4 \| Functional disturbances following cardiac surgery \| \| 458.2 \| Iatrogenic hypotension \| \| 458.21 \| Hypotension of haemodialysis \| \| 458.29 \| Other iatrogenic hypotension \| \| 512.1 \| Iatrogenic pneumothorax \| \| 512.2 \| Postoperative air leak \| \| 518.4 \| Acute oedema of lung unspecified \| \| 518.7 \| Transfusion related acute lung injury (TRALI) \| \| 519.0 \| Tracheostomy complications \| \| 519.00 \| Tracheostomy complication unspecified \| \| 519.01 \| Infection of tracheostomy \| \| 519.02 \| Mechanical complication of tracheostomy \| \| 519.09 \| Other tracheostomy complications \| \| 523.2 \| Gingival recession \| \| 530.86 \| Infection of oesophagostomy \| \| 530.87 \| Mechanical complication of oesophagostomy \| \| 536.4 \| Gastrostomy complications \| \| 536.40 \| Gastrostomy complication unspecified \| \| 536.41 \| Infection of gastrostomy \| \| 536.42 \| Mechanical complication of gastrostomy \| \| 536.49 \| Other gastrostomy complications \| \| 539.x \| Complications of bariatric procedures \| \| 551.21 \| Incisional ventral hernia with gangrene \| \| 553.21 \| Incisional hernia without obstruction or gangrene \| \| 560.81 \| Intestinal or peritoneal adhesions with obstruction (postoperative) (post infection) \| \| 564.2 \| Post gastric surgery syndromes \| \| 564.3 \| Vomiting following gastrointestinal surgery \| \| 564.4 \| Other postoperative functional disorders \| \| 568.0 \| Peritoneal adhesions (postoperative) (post infection) \| \| 569.6 \| Colostomy and enterostomy complications \| \| 569.62 \| Mechanical complication of colostomy and enterostomy \| \| 569.7x \| Other disorders of intestine: Complications of intestinal pouch \| \| 579.2 \| Blind loop syndrome \| \| 579.3 \| Other and unspecified postsurgical non absorption \| \| 593.3 \| Stricture or kinking of ureter \| \| 596.81 \| Infection of cystostomy \| \| 598.2 \| Postoperative urethral stricture \| \| 614.6 \| Pelvic peritoneal adhesions female (postoperative) (post infection) \| \| 728.13 \| Postoperative heterotopic calcification \| \| 780.62 \| Postprocedural fever \| \| 780.63 \| Postvaccination fever \| \| 780.66 \| Febrile nonhemolytic transfusion reaction \| \| 909.3 \| Late effect of complications of surgical and medical care \| \| 995.24 \| Failed moderate sedation during procedure \| \| 995.4 \| Shock due to anaesthesia \| \| 995.86 \| Malignant hyperthermia \| \| 997.x \| Complications affecting specified body system not elsewhere classified \| \| 997.99 \| Complications affecting other specified body systems not elsewhere classified \| \| 998.x \| Other complications of procedures not elsewhere classified \| \| 999.x \| Complications of medical care not elsewhere classified \| \| V15.53 \| Personal history of retained foreign body fully removed \| \| V15.80 \| Personal history of failed moderate sedation \| \| V15.83 \| Personal history of underimmunisation status \| \| V90.01 \| Retained depleted uranium fragments \| \| V90.09 \| Other retained radioactive fragments \| |  |  |
| --- | --- | --- | --- | --- | --- | --- | --- | --- | --- | --- | --- | --- | --- | --- | --- | --- | --- | --- | --- | --- | --- | --- | --- | --- | --- | --- | --- | --- | --- | --- | --- | --- | --- | --- | --- | --- | --- | --- | --- | --- | --- | --- | --- | --- | --- | --- | --- | --- | --- | --- | --- | --- | --- | --- | --- | --- | --- | --- | --- | --- | --- | --- | --- | --- | --- | --- | --- | --- | --- | --- | --- | --- | --- | --- | --- | --- | --- | --- | --- | --- | --- | --- | --- | --- | --- | --- | --- | --- | --- | --- | --- | --- | --- | --- | --- | --- | --- | --- | --- | --- | --- | --- | --- | --- | --- | --- | --- | --- | --- | --- | --- | --- | --- | --- | --- | --- | --- | --- | --- | --- | --- | --- | --- | --- | --- | --- | --- | --- | --- | --- | --- | --- | --- | --- | --- | --- | --- | --- | --- | --- | --- | --- | --- | --- | --- | --- | --- | --- | --- | --- | --- | --- | --- | --- | --- | --- | --- | --- | --- | --- | --- | --- | --- | --- | --- | --- | --- | --- | --- | --- | --- | --- | --- | --- | --- | --- | --- | --- | --- | --- | --- | --- | --- | --- | --- | --- | --- | --- | --- | --- | --- | --- | --- | --- | --- | --- | --- | --- | --- | --- | --- | --- | --- | --- | --- | --- | --- | --- | --- | --- | --- | --- | --- | --- | --- | --- | --- | --- | --- | --- | --- | --- | --- | --- | --- | --- | --- | --- | --- | --- | --- | --- | --- | --- | --- | --- | --- | --- | --- | --- | --- | --- | --- | --- | --- | --- | --- | --- | --- | --- | --- | --- | --- | --- | --- | --- | --- | --- | --- | --- | --- | --- | --- | --- | --- | --- | --- | --- | --- | --- | --- | --- | --- | --- | --- | --- | --- | --- | --- | --- | --- | --- | --- | --- | --- | --- | --- | --- | --- | --- | --- | --- | --- | --- | --- | --- | --- | --- | --- | --- | --- | --- | --- | --- | --- | --- | --- | --- | --- | --- | --- | --- | --- | --- | --- | --- | --- | --- | --- | --- | --- | --- | --- | --- | --- | --- | --- | --- | --- | --- | --- | --- | --- | --- | --- | --- | --- | --- | --- | --- | --- | --- | --- | --- | --- | --- | --- | --- | --- | --- | --- | --- | --- | --- | --- | --- | --- | --- | --- | --- | --- | --- | --- | --- | --- | --- | --- | --- | --- | --- | --- | --- | --- | --- | --- | --- | --- | --- | --- | --- | --- | --- | --- | --- | --- | --- | --- | --- | --- | --- | --- | --- | --- | --- | --- | --- | --- | --- | --- | --- | --- | --- | --- | --- | --- | --- | --- | --- | --- | --- | --- | --- | --- | --- | --- | --- | --- | --- | --- | --- | --- | --- | --- | --- | --- | --- | --- | --- | --- | --- | --- | --- | --- | --- | --- | --- | --- | --- | --- | --- | --- | --- | --- | --- | --- | --- | --- | --- | --- | --- | --- | --- | --- | --- | --- | --- | --- | --- | --- | --- | --- | --- | --- | --- | --- | --- | --- | --- | --- | --- | --- | --- | --- | --- | --- | --- | --- | --- | --- | --- | --- | --- | --- | --- | --- | --- | --- | --- | --- | --- | --- | --- | --- | --- | --- | --- | --- | --- | --- | --- | --- | --- | --- | --- | --- | --- | --- | --- | --- | --- | --- | --- | --- | --- | --- | --- | --- | --- | --- | --- | --- | --- | --- | --- | --- | --- | --- | --- | --- | --- | --- | --- | --- | --- | --- | --- | --- | --- | --- | --- | --- | --- | --- | --- | --- | --- | --- | --- | --- | --- | --- | --- | --- | --- | --- | --- | --- | --- | --- | --- | --- | --- | --- | --- | --- | --- | --- | --- | --- | --- | --- | --- | --- | --- | --- | --- | --- | --- | --- | --- | --- | --- | --- | --- | --- | --- | --- | --- | --- | --- | --- | --- | --- | --- |
|  |  |  |

**Supplementary Table S1.** Multivariate (adjusted) Cox proportional hazards analyses exploring the effect on outcomes of the implementation of the primary-care hospital integrated heart failure programme according to age group strata in the HUB-Delta healthcare area between 1 January 2015 and 31 December 2019. Models were adjusted for sex, SES, previous hospitalisation, morbidity index (GMA: associated morbidity groups) and time since diagnosis of HF.

|  |  | **HUB-Delta vs rest of Catalonia** | | |
| --- | --- | --- | --- | --- |
| **Age** | **Period** | **HR** | **CI 95%** | **p-value** |
| **All-cause mortality** | | | | |
| 15-74 years | 2015 | 1.10 | 0.92-1.33 | 0.304 |
|  | 2016 | 1.17 | 0.95-1.44 | 0.144 |
|  | 2017 | 0.94 | 0.72-1.23 | 0.643 |
|  | 2018 | 0.60 | 0.40-0.91 | 0.015 |
|  | 2019 | 0.90 | 0.48-1.69 | 0.745 |
| 75-84 years | 2015 | 0.92 | 0.81-1.04 | 0.169 |
|  | 2016 | 0.77 | 0.66-0.89 | 0.001 |
|  | 2017 | 0.92 | 0.78-1.09 | 0.336 |
|  | 2018 | 0.86 | 0.68-1.09 | 0.212 |
|  | 2019 | 0.83 | 0.55-1.26 | 0.380 |
| >84 years | 2015 | 1.02 | 0.89-1.16 | 0.817 |
|  | 2016 | 0.95 | 0.82-1.11 | 0.534 |
|  | 2017 | 0.91 | 0.78-1.07 | 0.261 |
|  | 2018 | 0.86 | 0.72-1.03 | 0.092 |
|  | 2019 | 0.72 | 0.53-0.98 | 0.035 |
| **Clinically-related hospitalisation** | | | | |
| 15-74 years | 2015 | 1.20 | 1.04-1.40 | 0.016 |
|  | 2016 | 1.24 | 1.06-1.46 | 0.008 |
|  | 2017 | 1.25 | 1.05-1.49 | 0.014 |
|  | 2018 | 0.81 | 0.65-1.02 | 0.070 |
|  | 2019 | 0.64 | 0.43-0.95 | 0.026 |
| 75-84 years | 2015 | 1.25 | 1.11-1.40 | 0.000 |
|  | 2016 | 1.08 | 0.95-1.23 | 0.265 |
|  | 2017 | 1.13 | 0.98-1.31 | 0.083 |
|  | 2018 | 0.83 | 0.68-1.00 | 0.051 |
|  | 2019 | 0.78 | 0.57-1.07 | 0.129 |
| >84 years | 2015 | 1.29 | 1.10-1.50 | 0.001 |
|  | 2016 | 1.42 | 1.22-1.67 | 0.000 |
|  | 2017 | 1.17 | 0.98-1.39 | 0.083 |
|  | 2018 | 1.03 | 0.85-1.23 | 0.796 |
|  | 2019 | 1.08 | 0.84-1.40 | 0.553 |
| **HF readmission** | | | | |
| 15-74 years | 2015 | 1.28 | 1.07-1.53 | 0.007 |
|  | 2016 | 1.35 | 1.12-1.63 | 0.002 |
|  | 2017 | 1.37 | 1.10-1.69 | 0.004 |
|  | 2018 | 0.57 | 0.41-0.80 | 0.001 |
|  | 2019 | 0.67 | 0.40-1.12 | 0.124 |
| 75-84 years | 2015 | 1.33 | 1.17-1.52 | 0.000 |
|  | 2016 | 1.31 | 1.13-1.51 | 0.000 |
|  | 2017 | 1.24 | 1.06-1.46 | 0.009 |
|  | 2018 | 0.80 | 0.63-1.01 | 0.055 |
|  | 2019 | 0.56 | 0.37-0.87 | 0.009 |
| >84 years | 2015 | 1.32 | 1.11-1.56 | 0.002 |
|  | 2016 | 1.65 | 1.40-1.95 | 0.000 |
|  | 2017 | 1.25 | 1.03-1.52 | 0.022 |
|  | 2018 | 1.08 | 0.88-1.33 | 0.445 |
|  | 2019 | 1.12 | 0.83-1.50 | 0.465 |

**SUPPLEMENTARY FIGURES**

**Supplementary Figure S1.** Survival curves estimated on the basis of multivariate (adjusted) Cox models evaluating the impact on adjusted probability of all-cause mortality according to healthcare setting (HUB-Delta vs. Rest of Catalonia) across predefined periods: 2015-2016 (panel A), 2017 (panel B) and 2018-2019 (panel C) in the age group 15-74.

A)


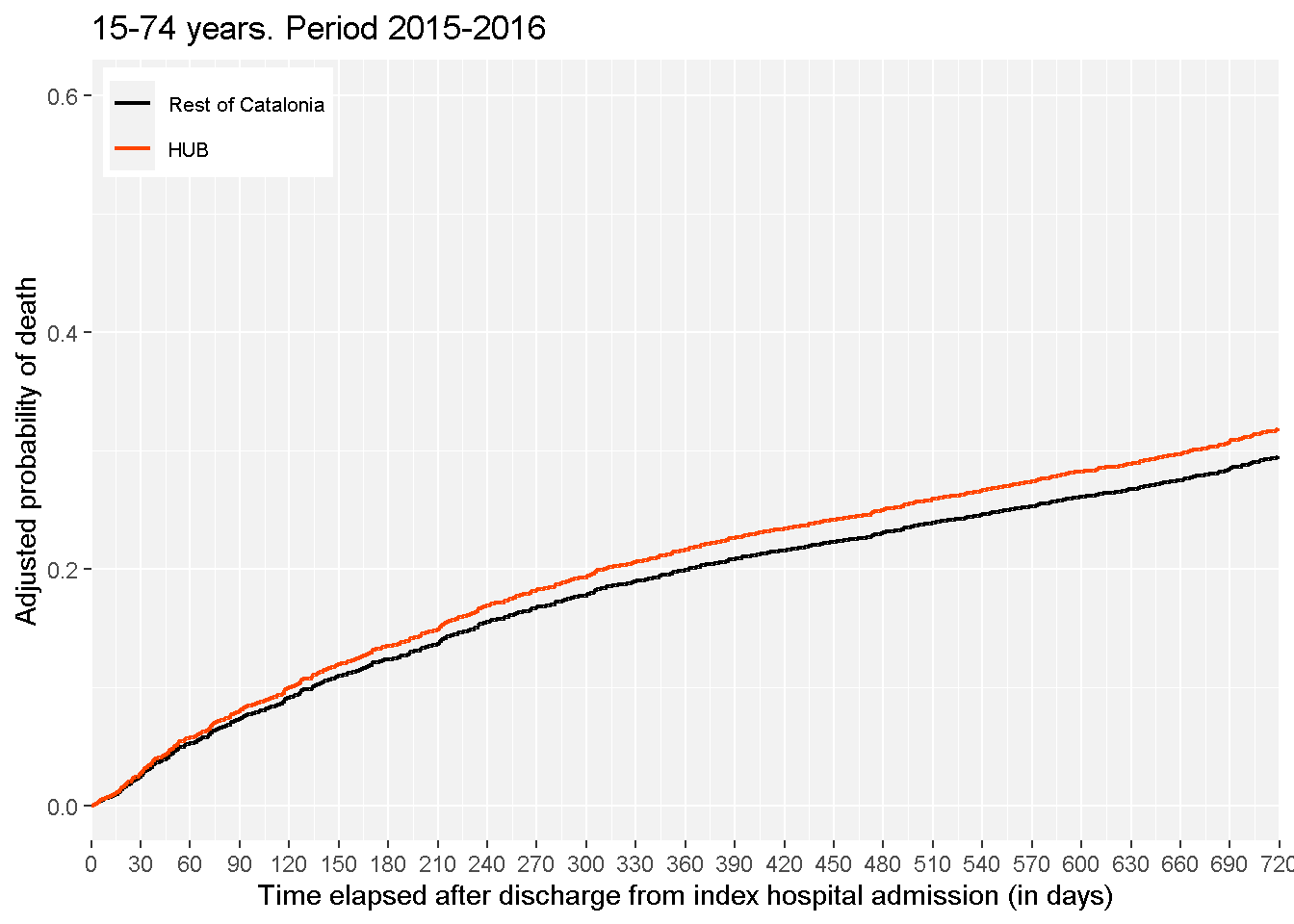


B)


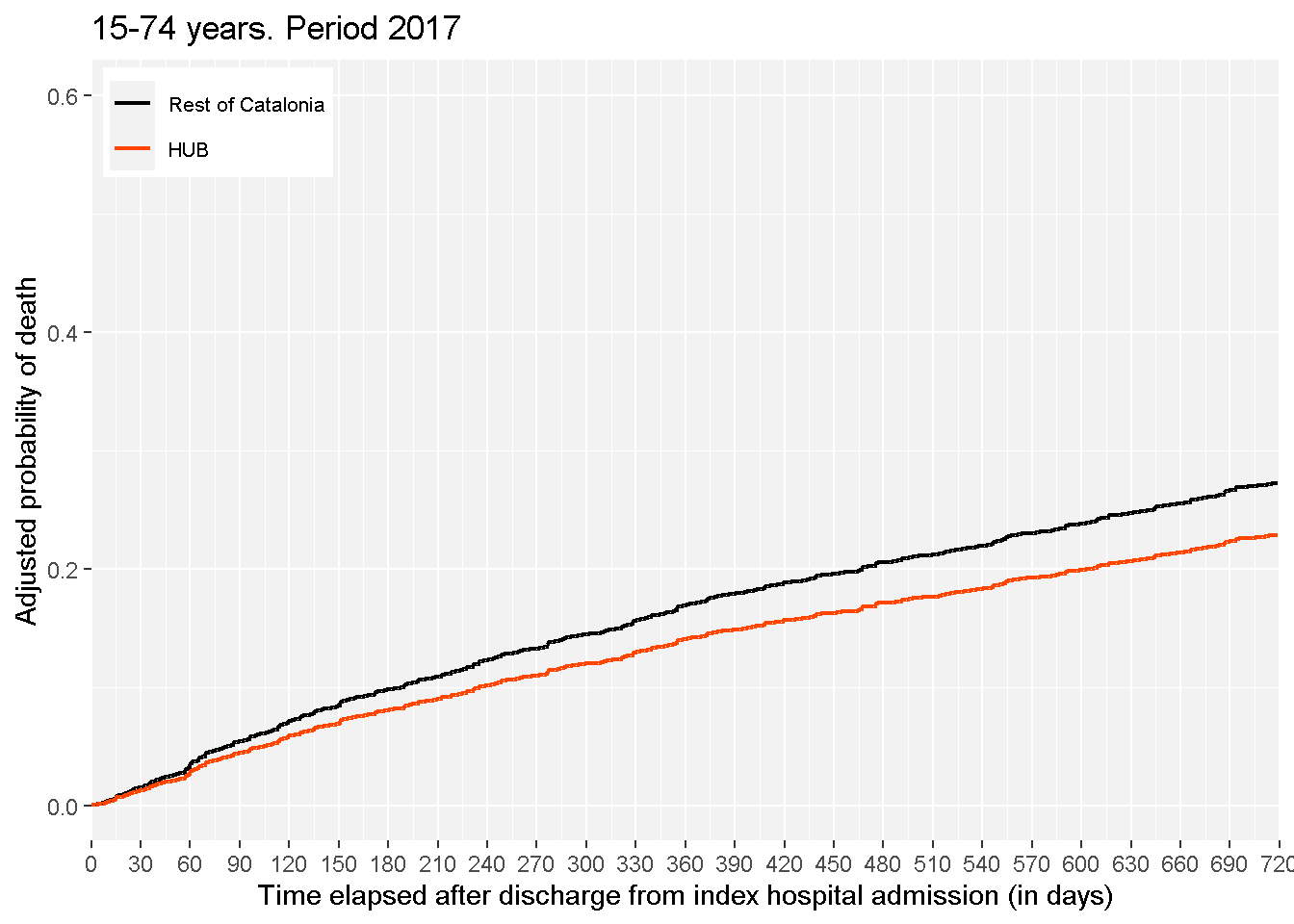


C)


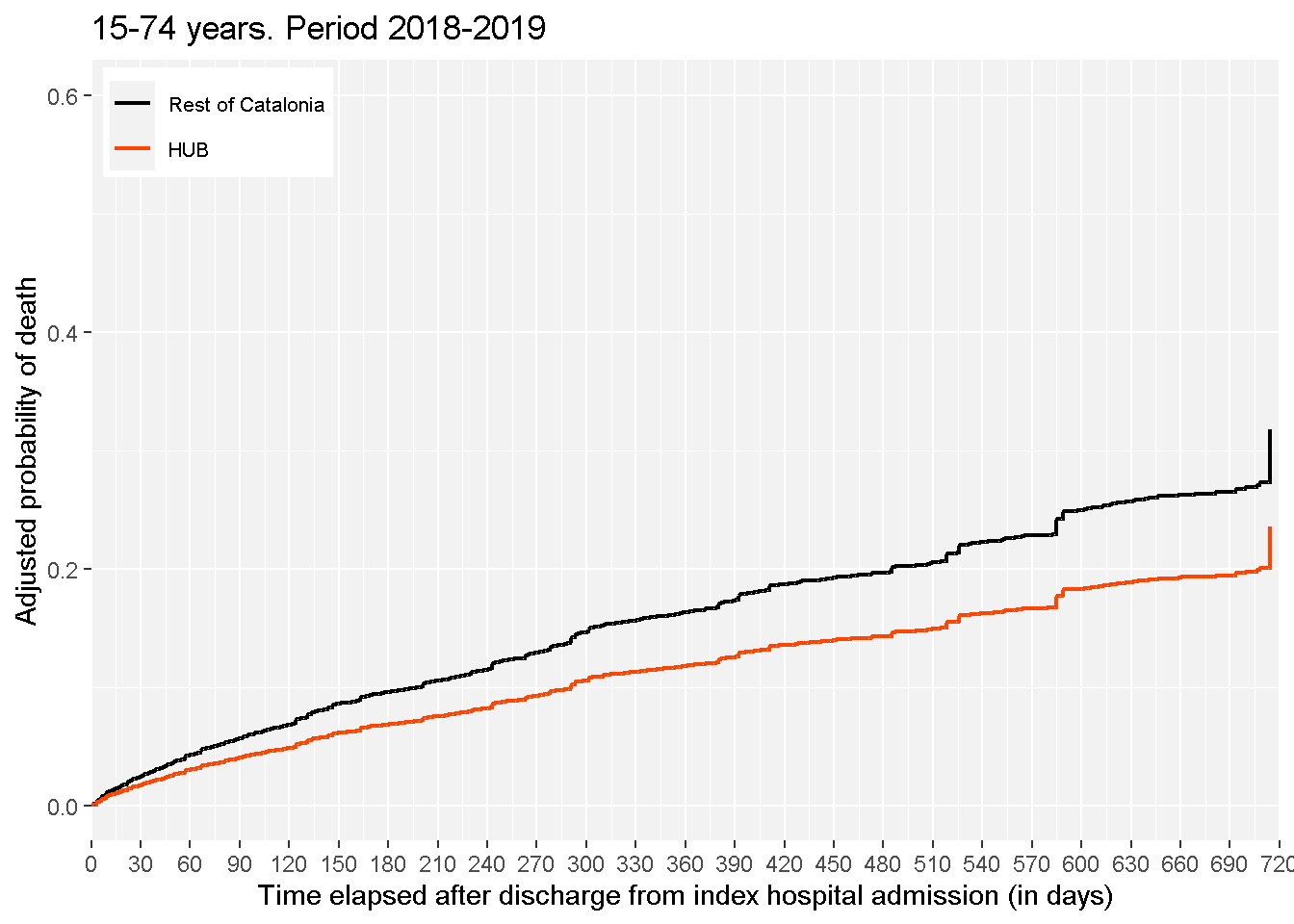


**Supplementary Figure S2**. Survival curves estimated on the basis of multivariate (adjusted) Cox models evaluating the impact on adjusted probability of all-cause mortality according to healthcare setting (HUB-Delta vs. Rest of Catalonia) across predefined periods: 2015-2016 (panel A), 2017 (panel B) and 2018-2019 (panel C) in the age group 75-84.

A)


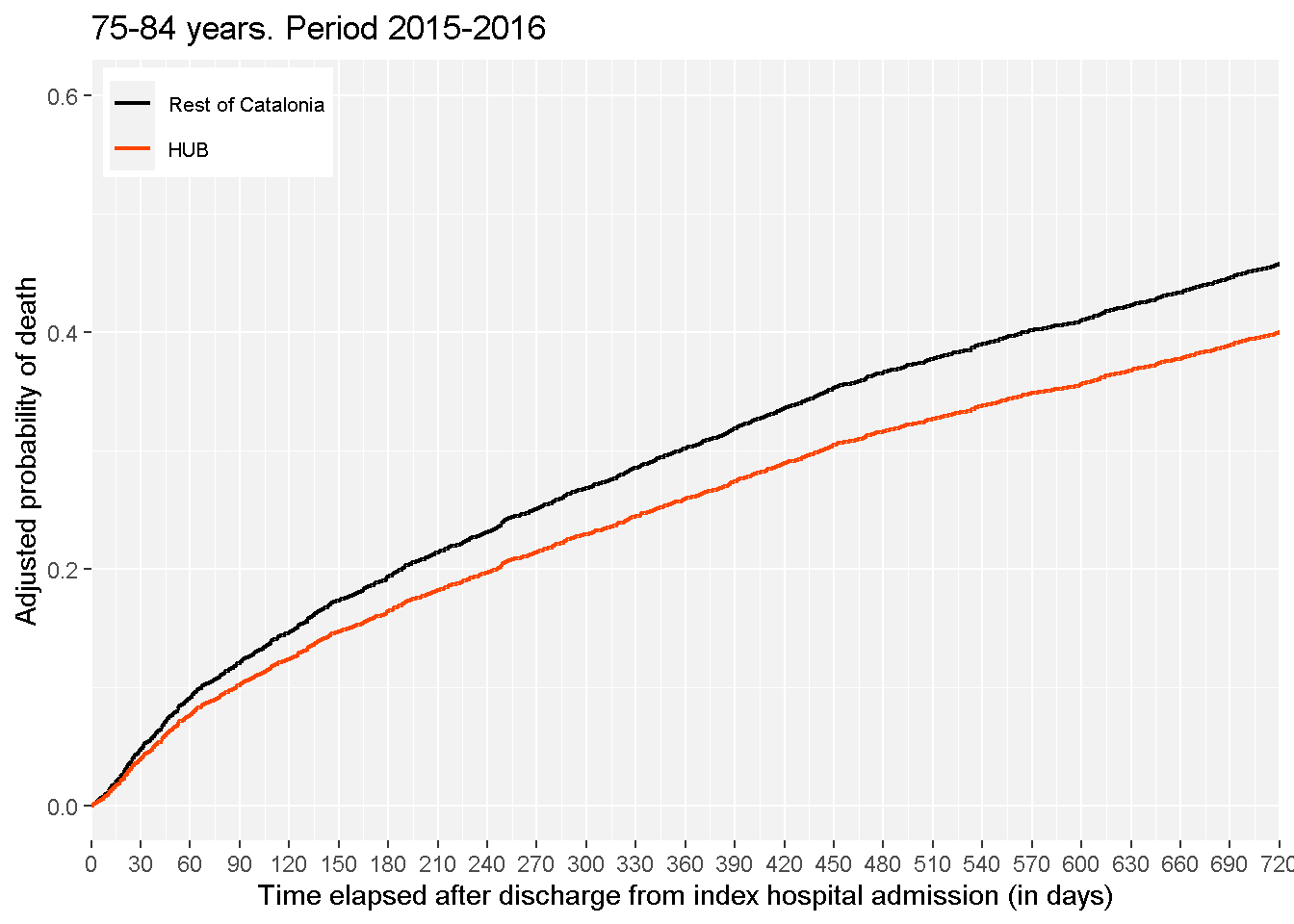


B)


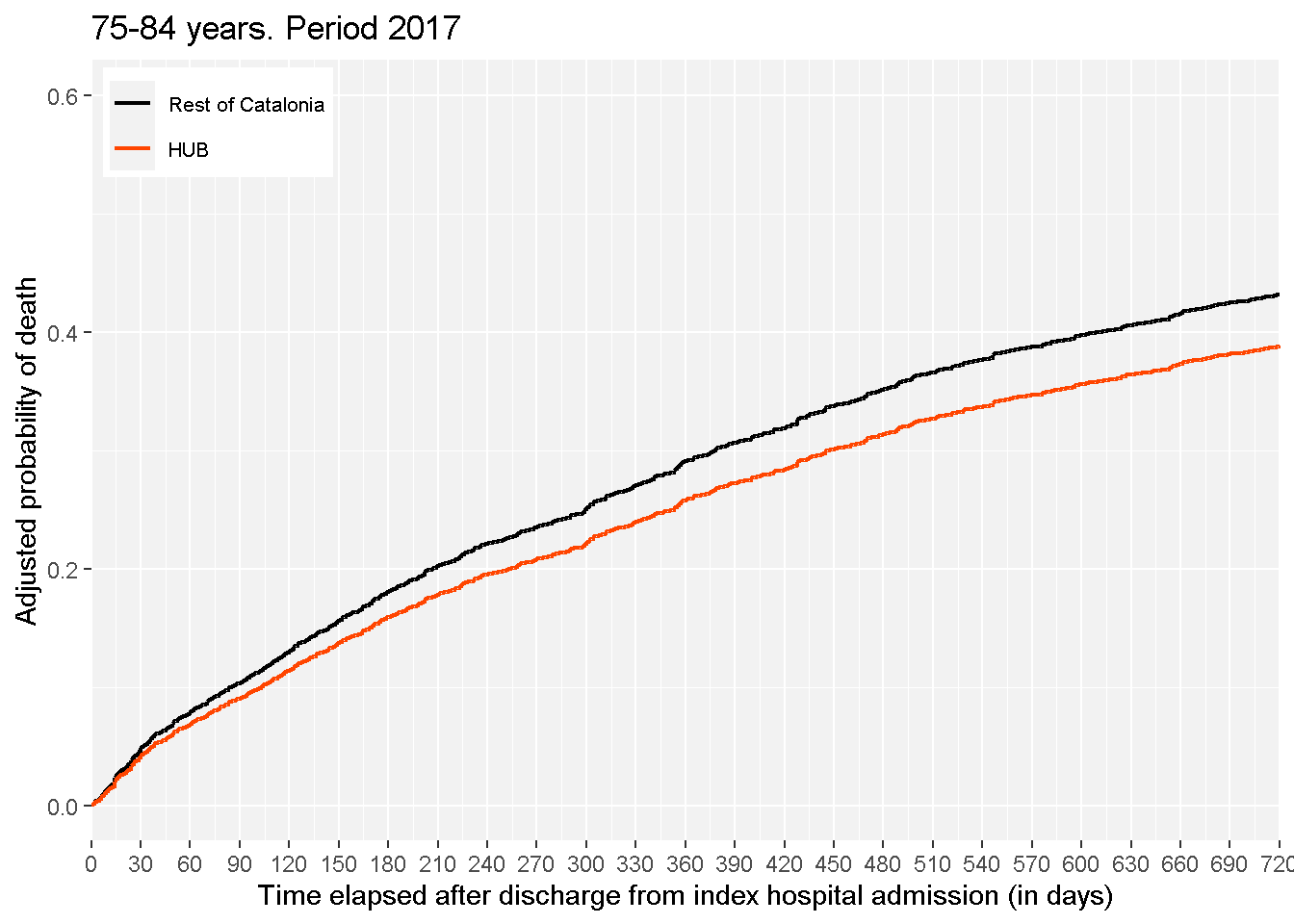


C)


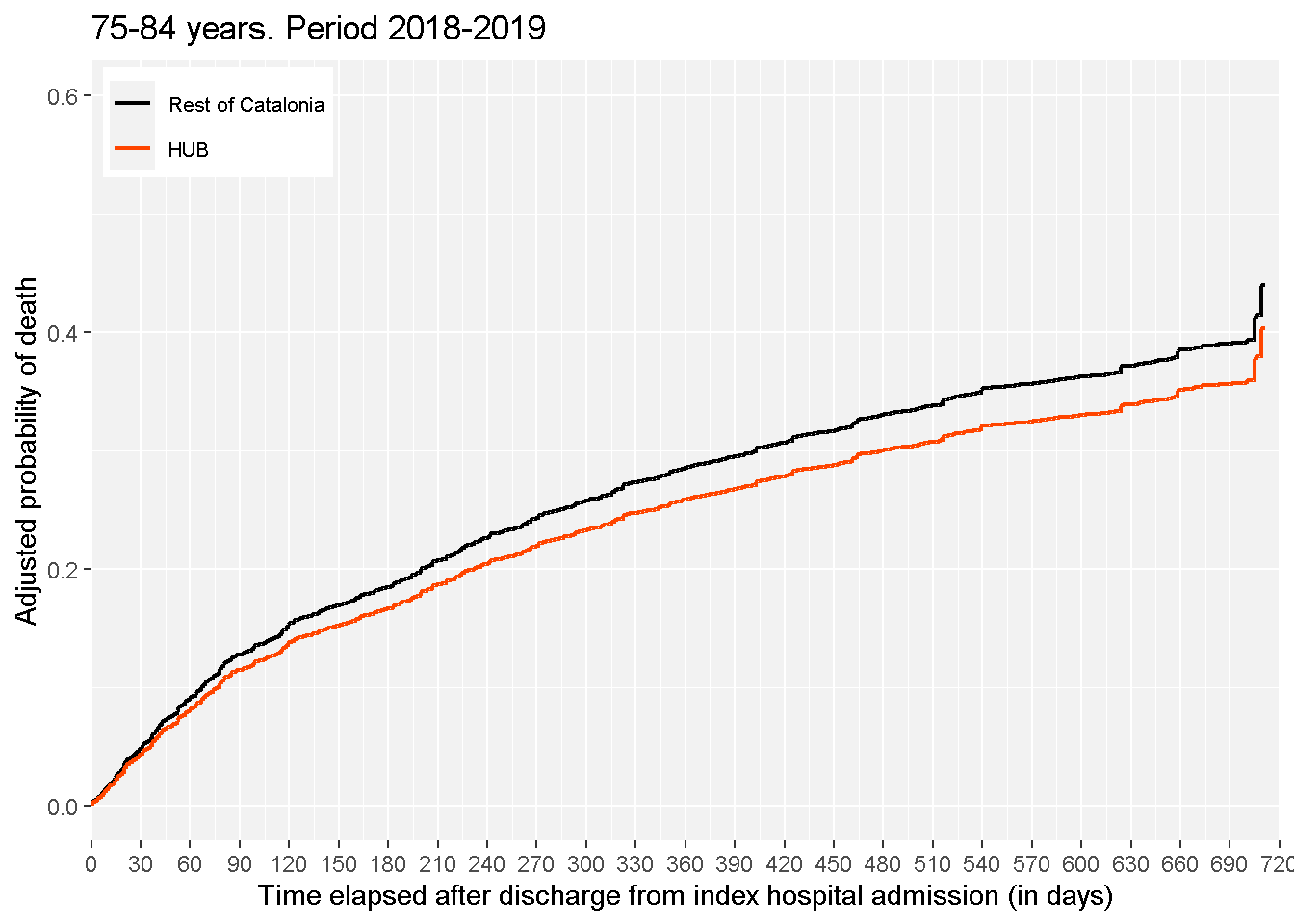


**Supplementary Figure S3**. Survival curves estimated on the basis of multivariate (adjusted) Cox models evaluating the impact on adjusted probability of all-cause mortality according to healthcare setting (HUB-Delta vs. Rest of Catalonia) across predefined periods: 2015-2016 (panel A), 2017 (panel B) and 2018-2019 (panel C) in the age group >84.

A)


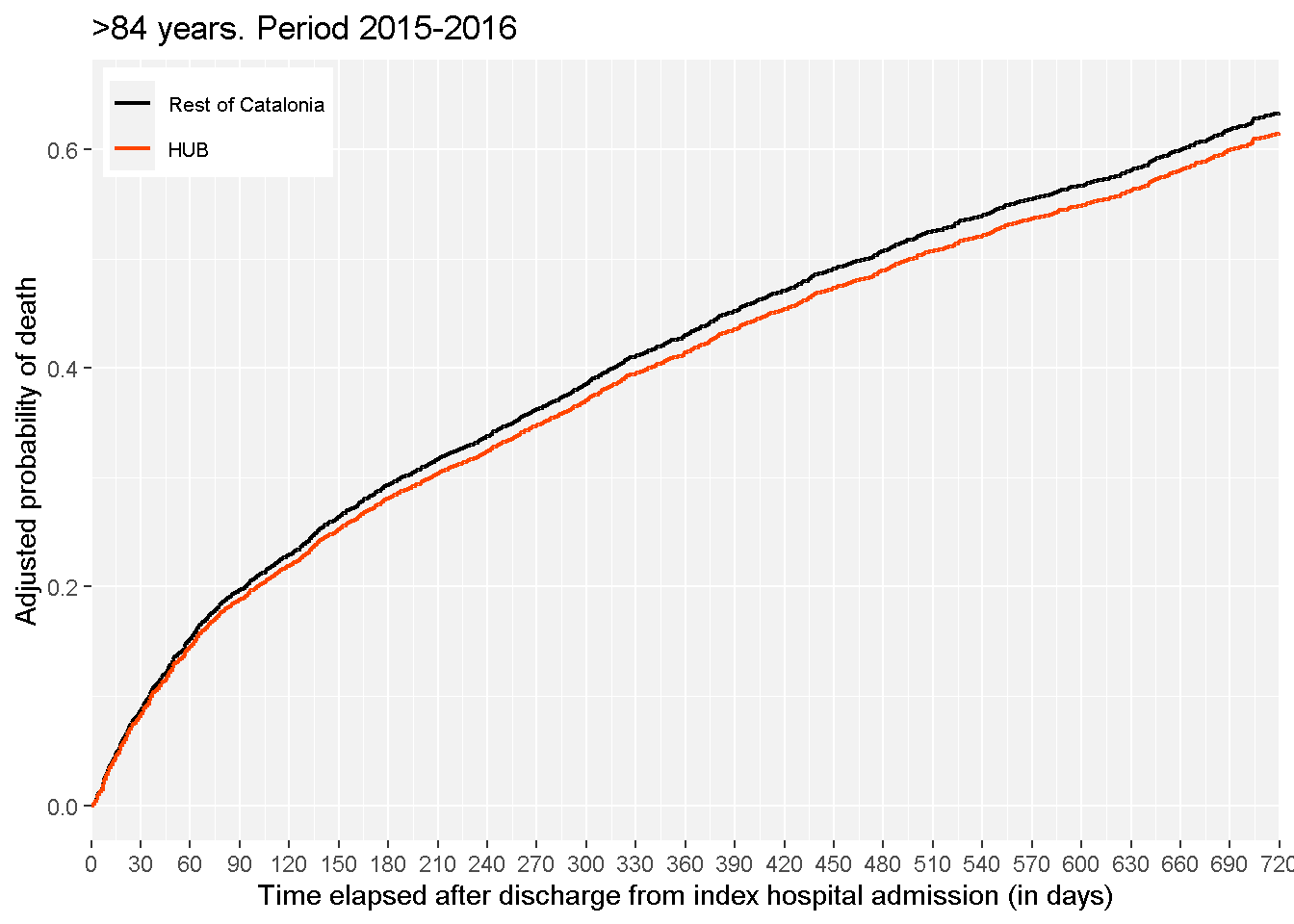


B)


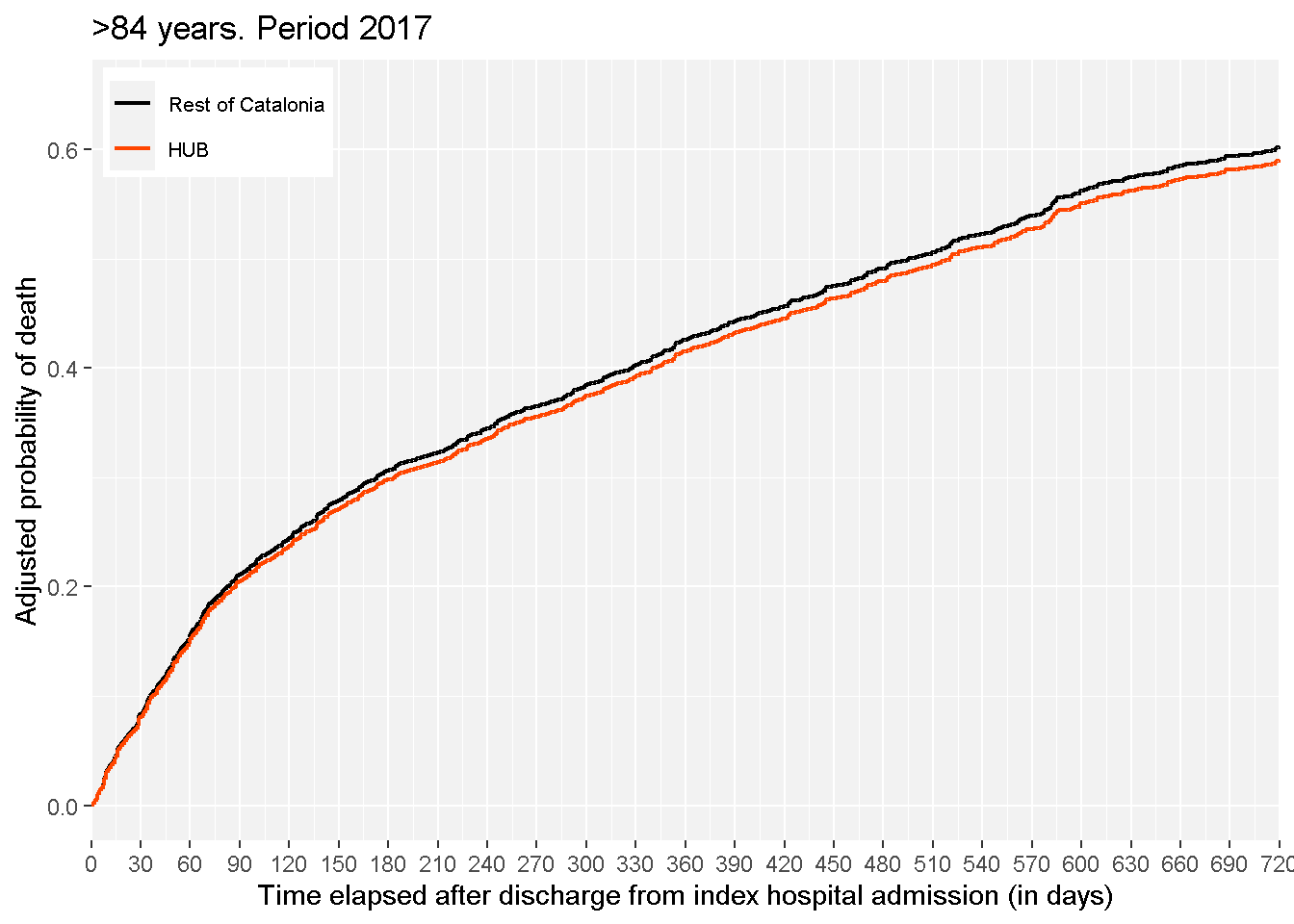


C)


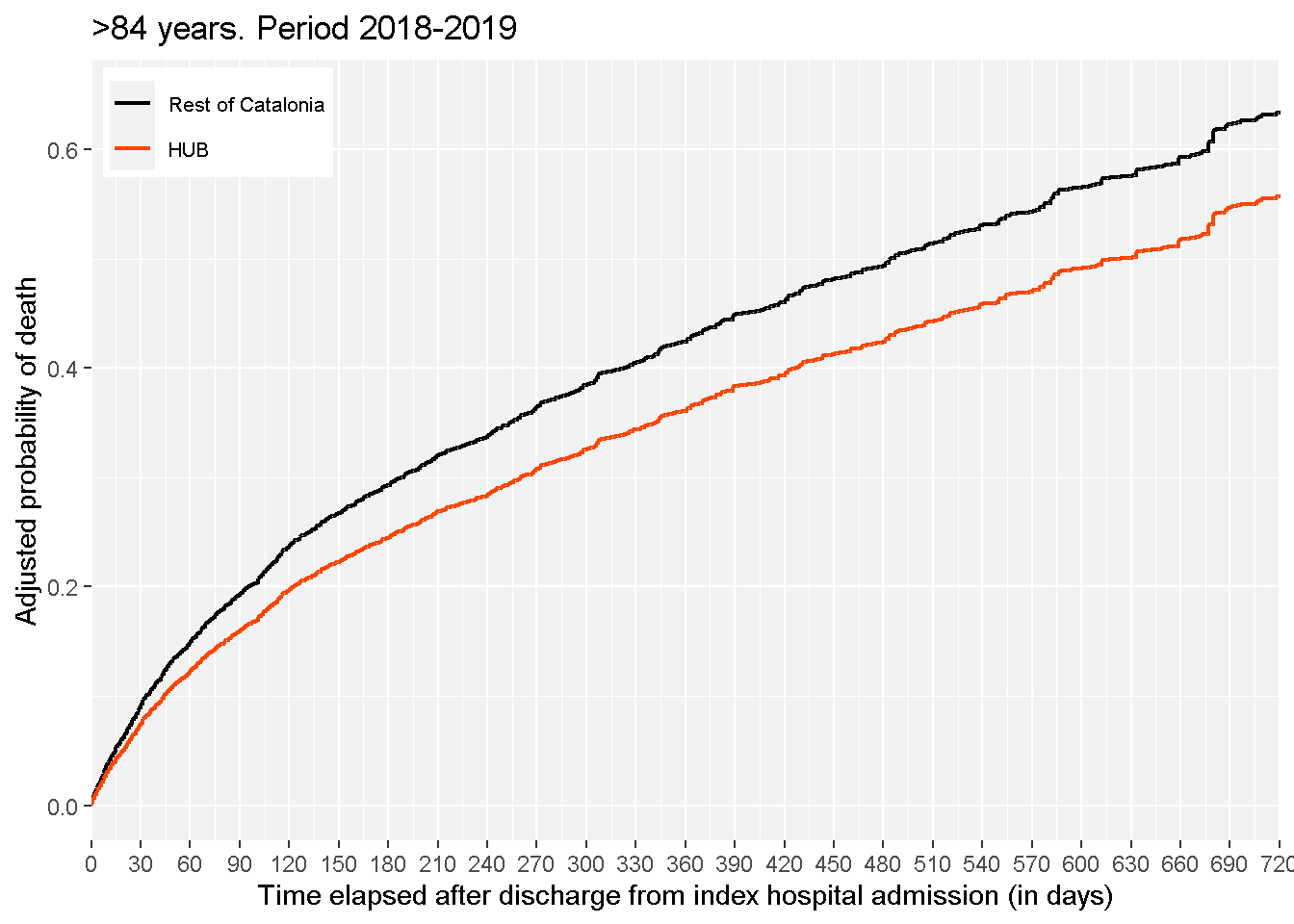


**Supplementary Figure S4.** Survival curves estimated on the basis of multivariate (adjusted) Cox models evaluating the impact on adjusted probability of clinically-related readmission according to healthcare setting (HUB-Delta vs. Rest of Catalonia) across predefined periods: 2015-2016 (panel A), 2017 (panel B) and 2018-2019 (panel C) in the age group 15-74.

A)


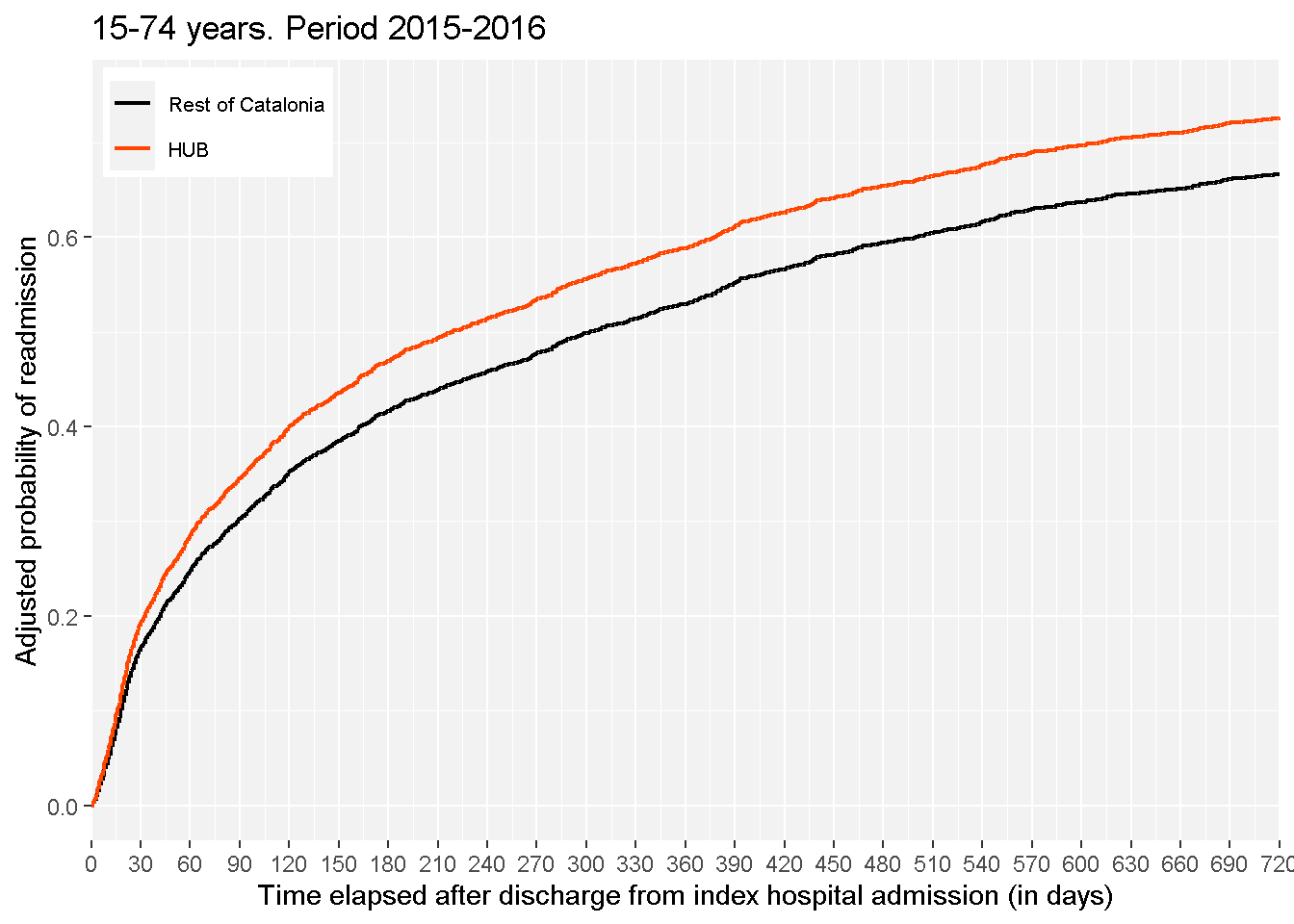


B)


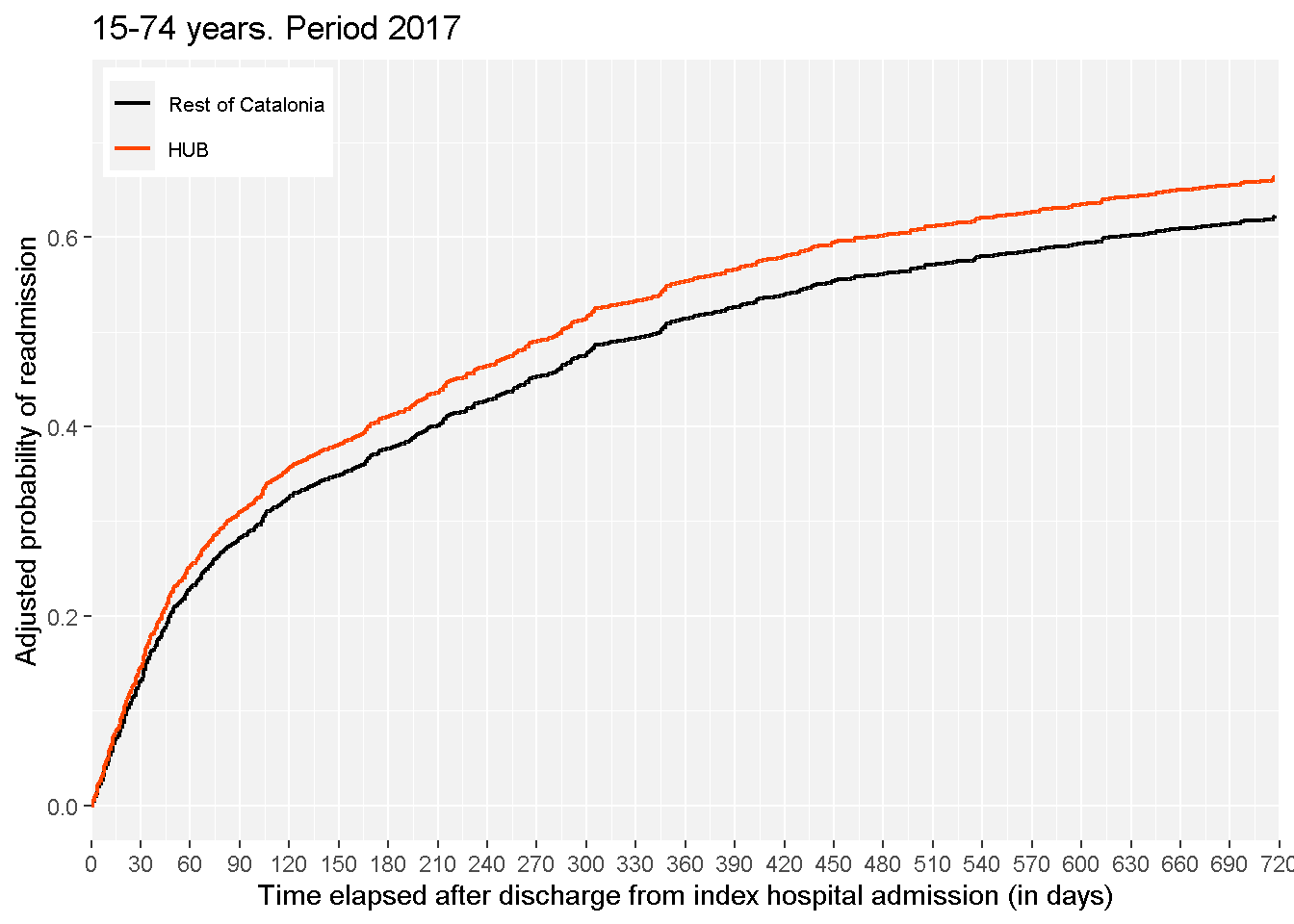


C)


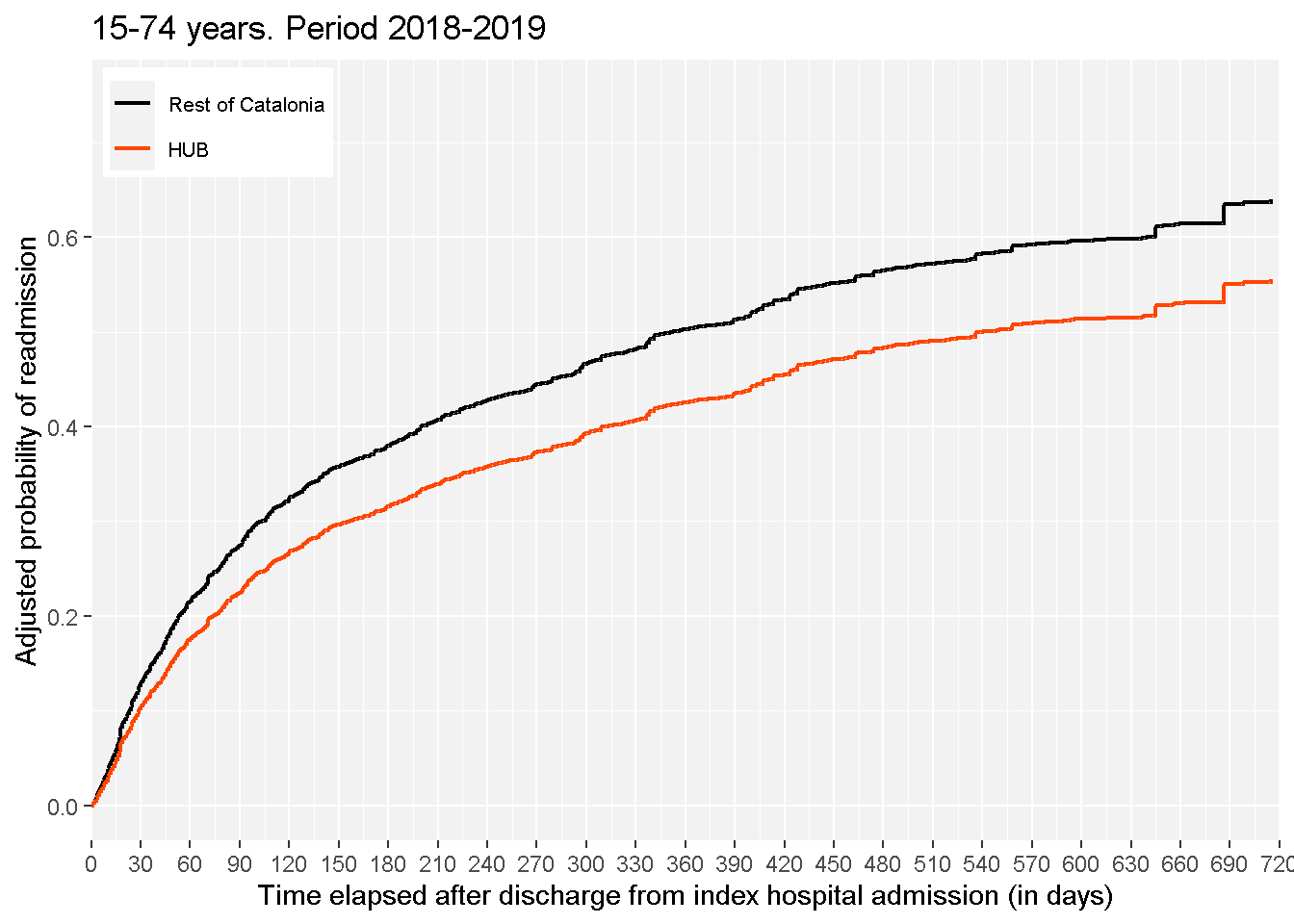


**Supplementary Figure S5.** Survival curves estimated on the basis of multivariate (adjusted) Cox models evaluating the impact on adjusted probability of clinically-related readmission according to healthcare setting (HUB-Delta vs. Rest of Catalonia) across predefined periods: 2015-2016 (panel A), 2017 (panel B) and 2018-2019 (panel C) in the age group 75-84.

A)
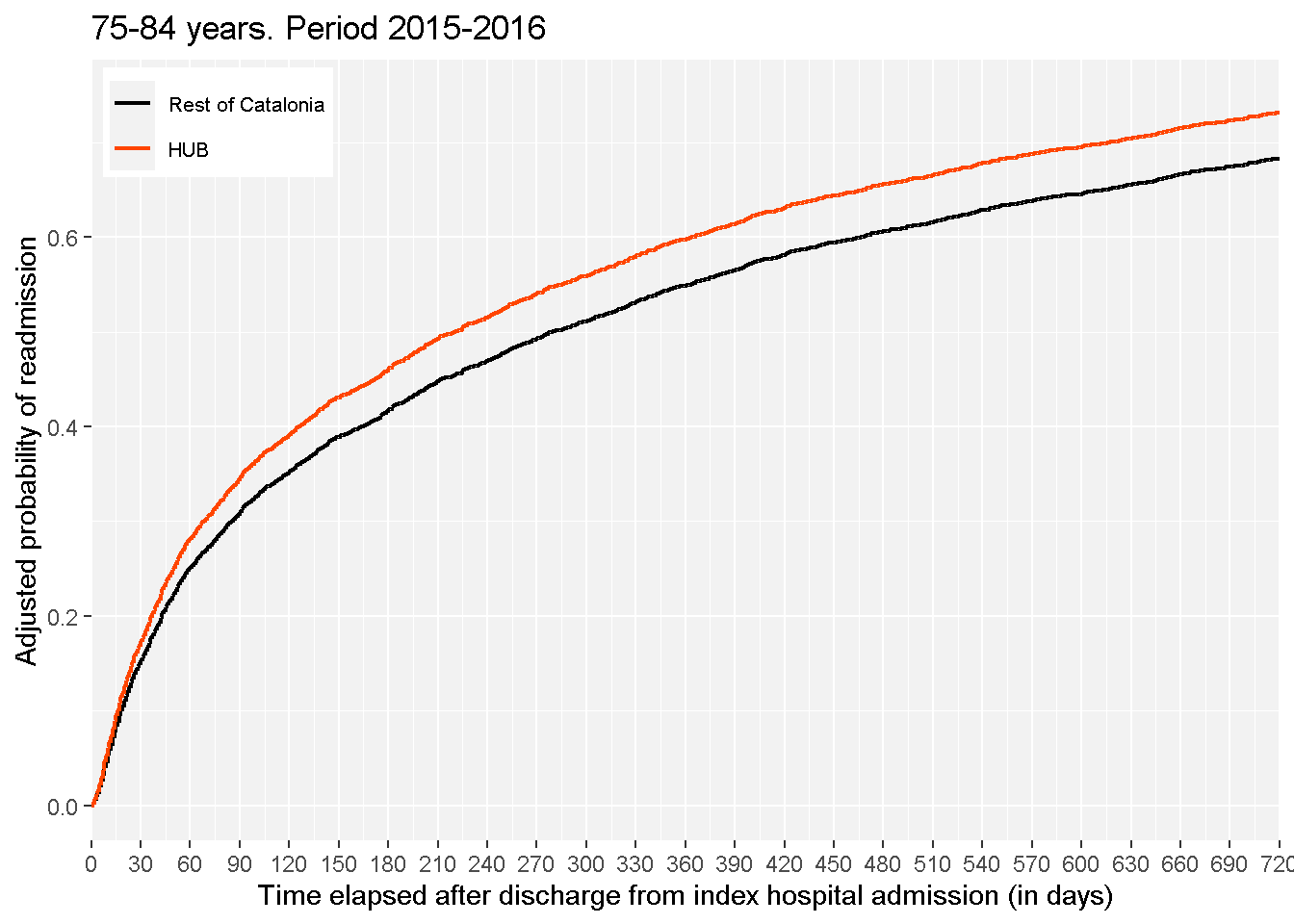


B)


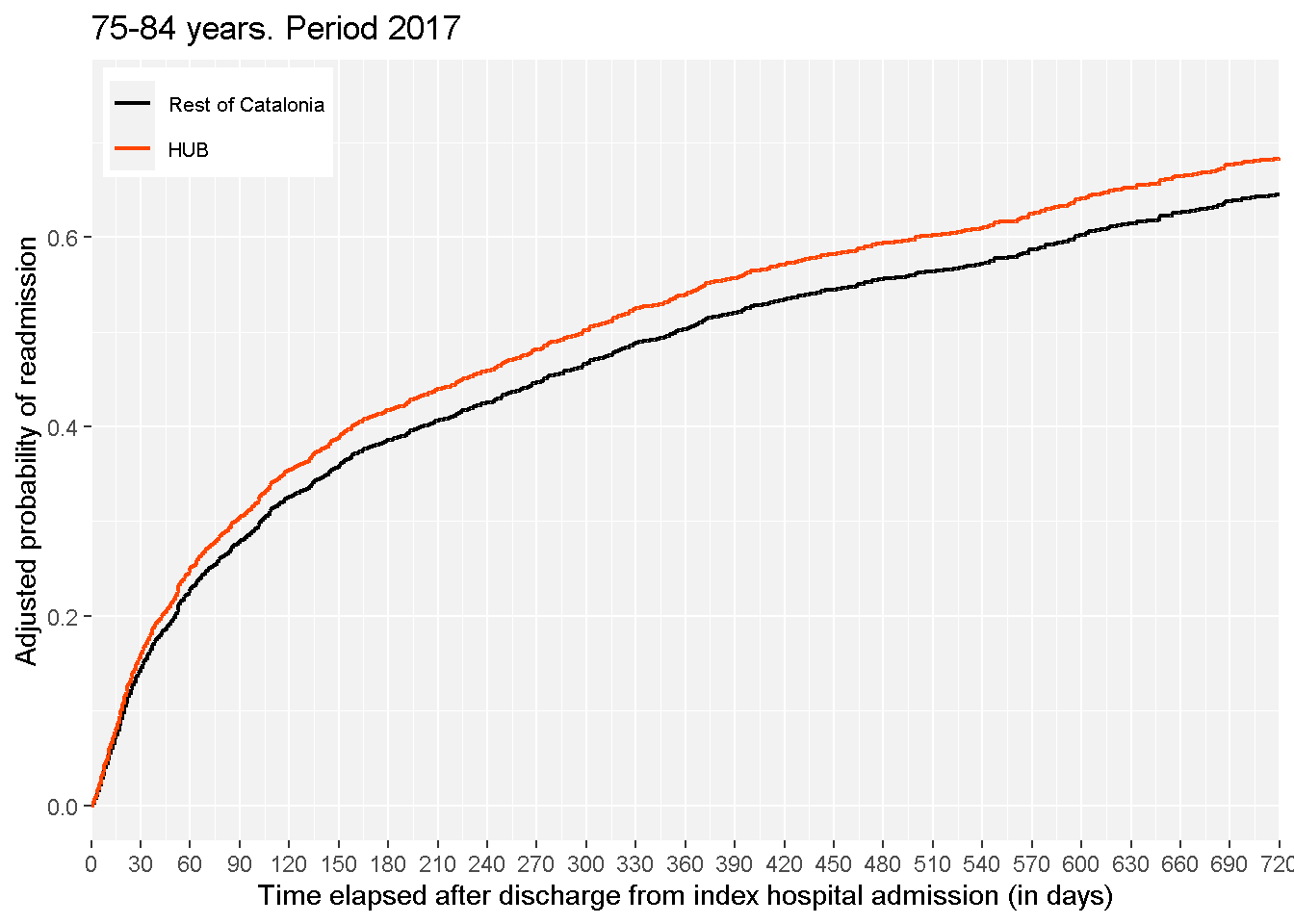


C)


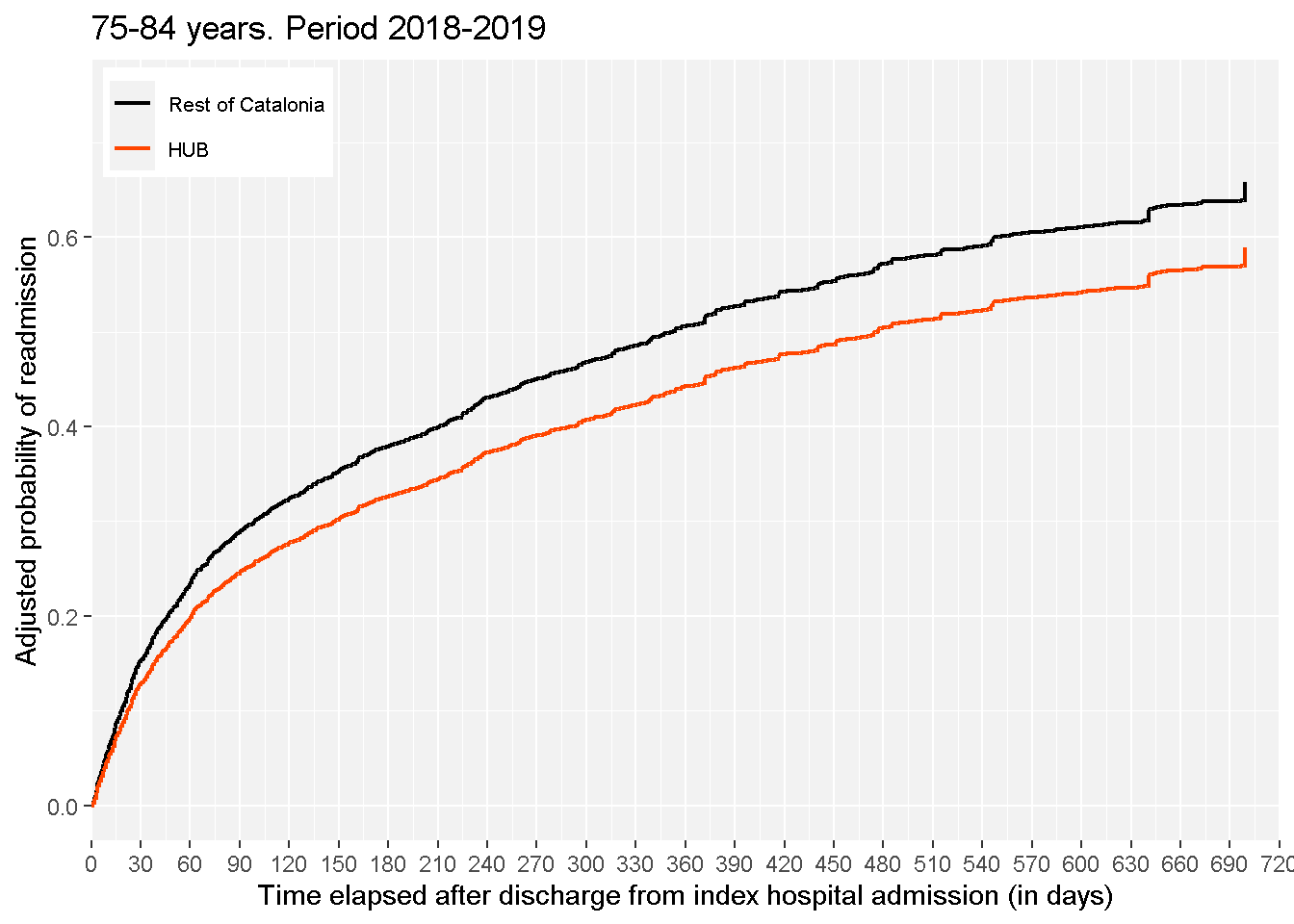


**Supplementary Figure S6.** Survival curves estimated on the basis of multivariate (adjusted) Cox models evaluating the impact on adjusted probability of clinically-related readmission according to healthcare setting (HUB-Delta vs. Rest of Catalonia) across predefined periods: 2015-2016 (panel A), 2017 (panel B) and 2018-2019 (panel C) in the age group >84.

A)


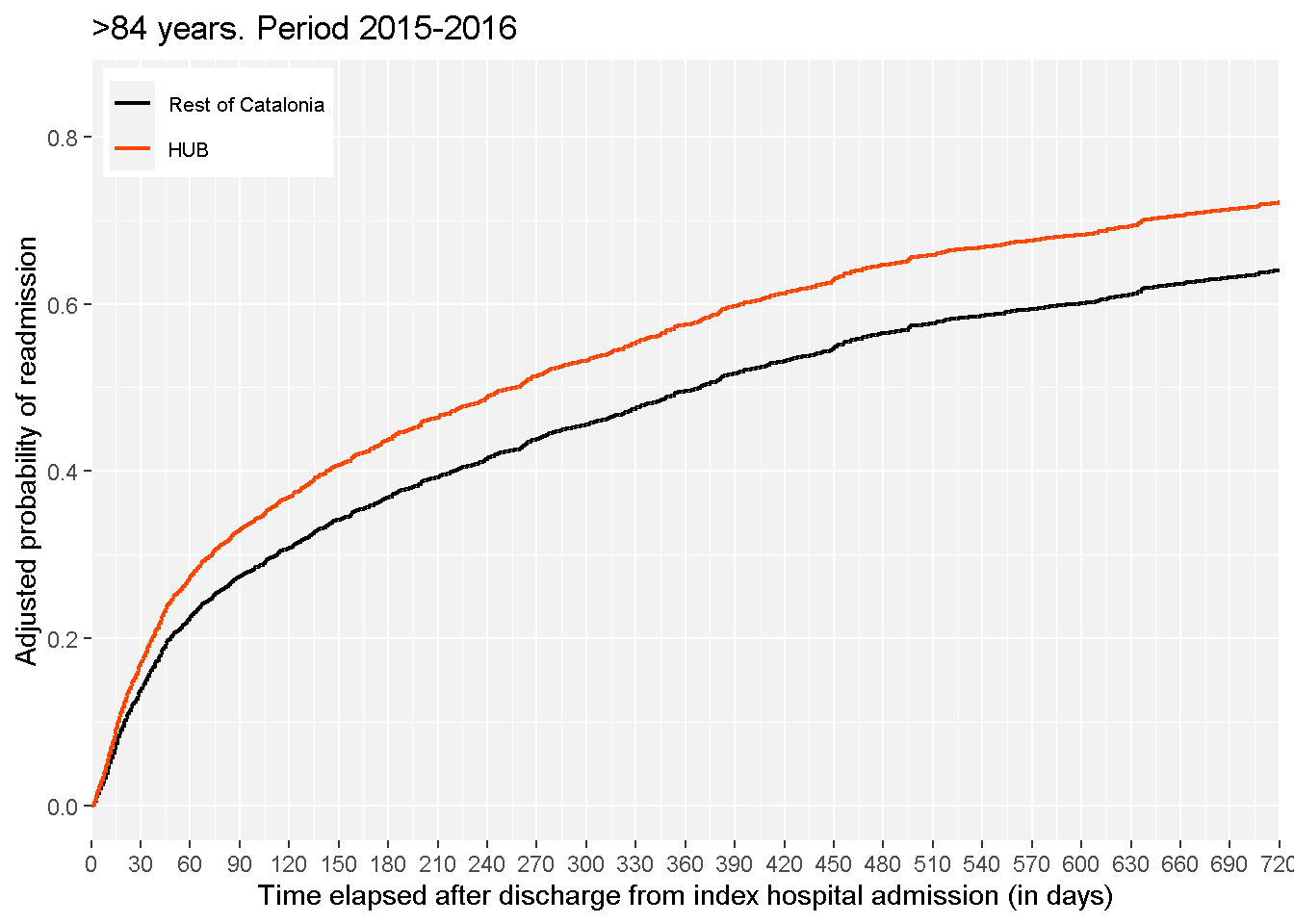


B)
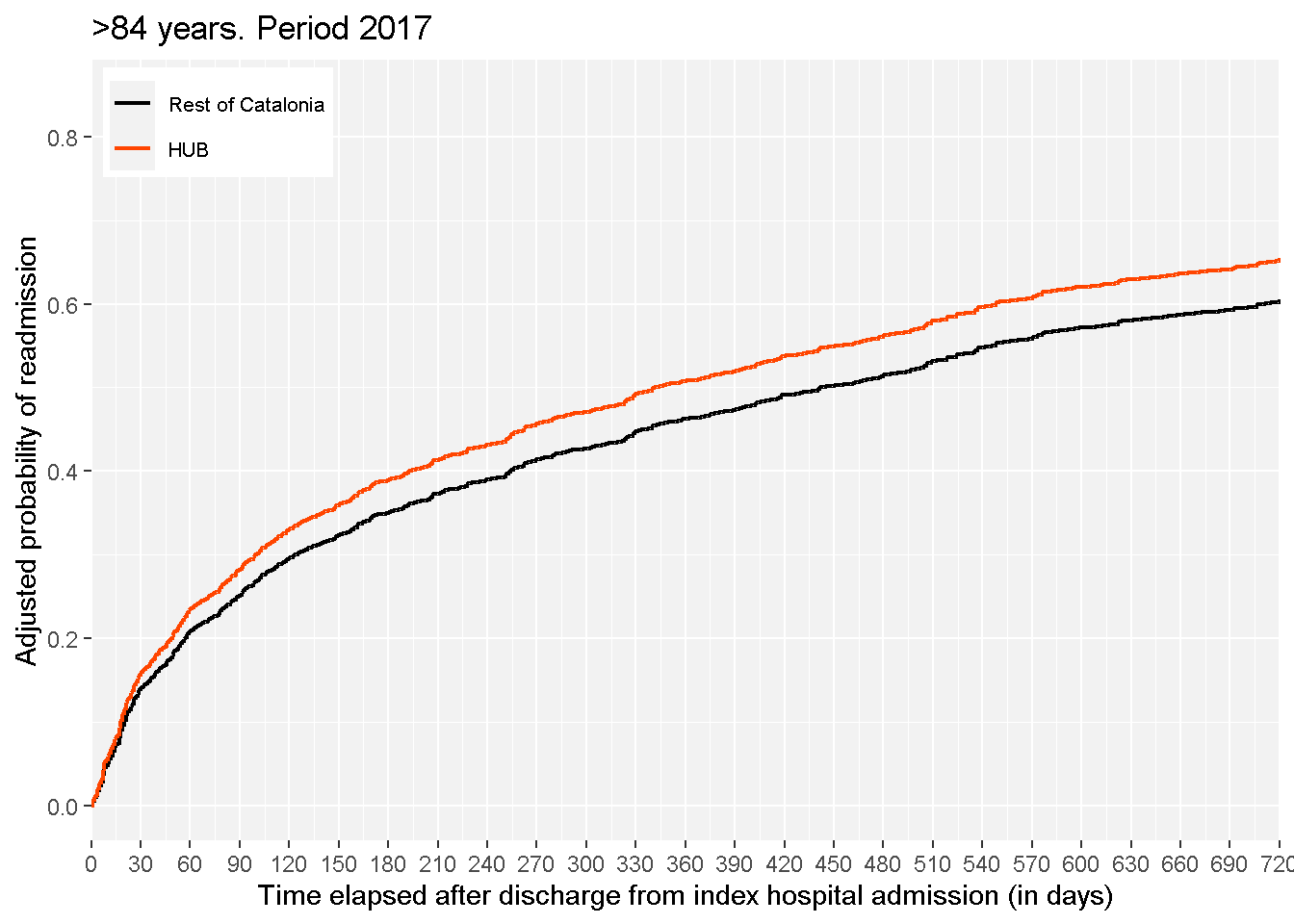


C)


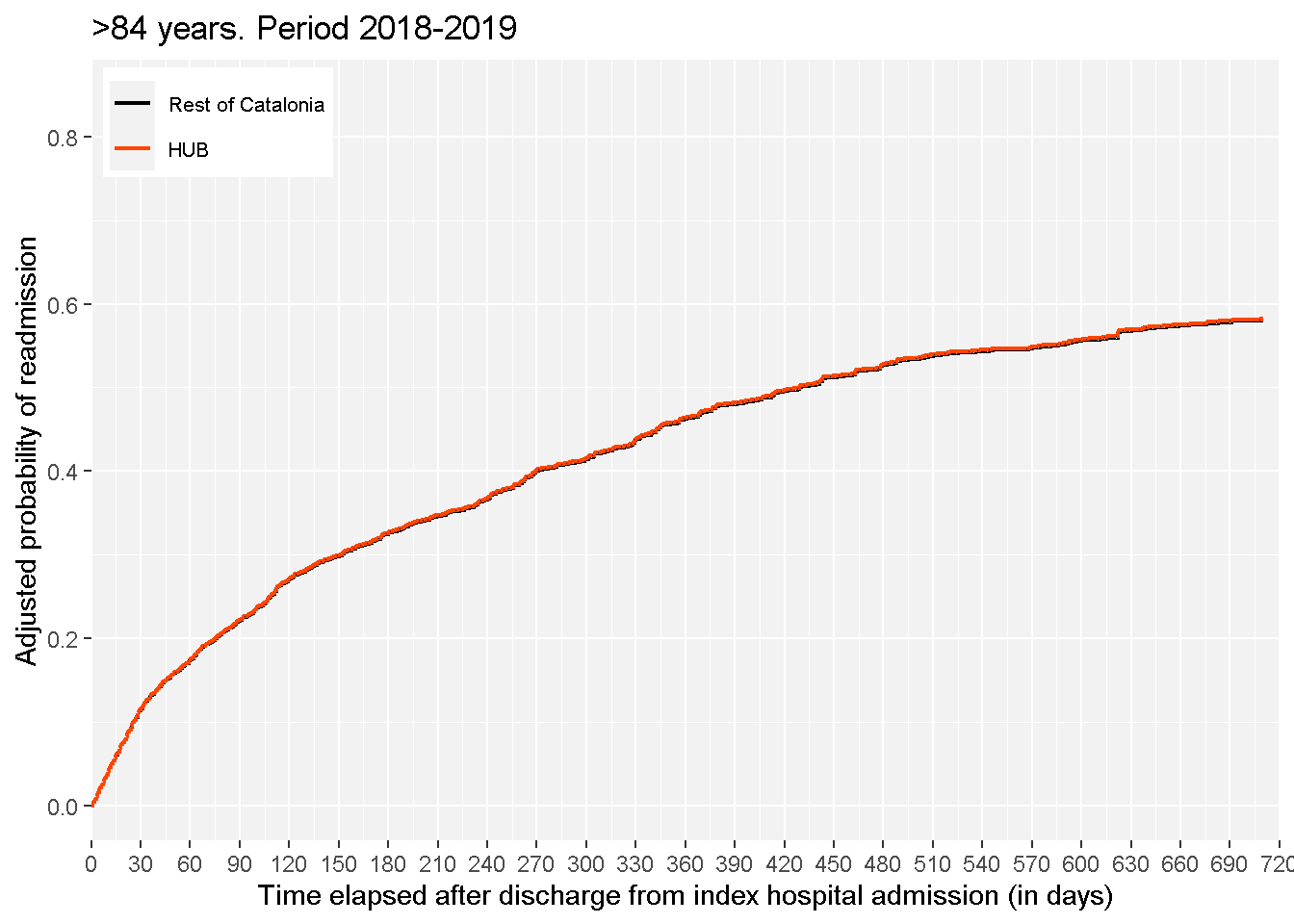


**Supplementary Figure S7.** Survival curves estimated on the basis of multivariate (adjusted) Cox models evaluating the impact on adjusted probability of HF readmission according to healthcare setting (HUB-Delta vs. Rest of Catalonia) across predefined periods: 2015-2016 (panel A), 2017 (panel B) and 2018-2019 (panel C) in the age group 15-74.

A)
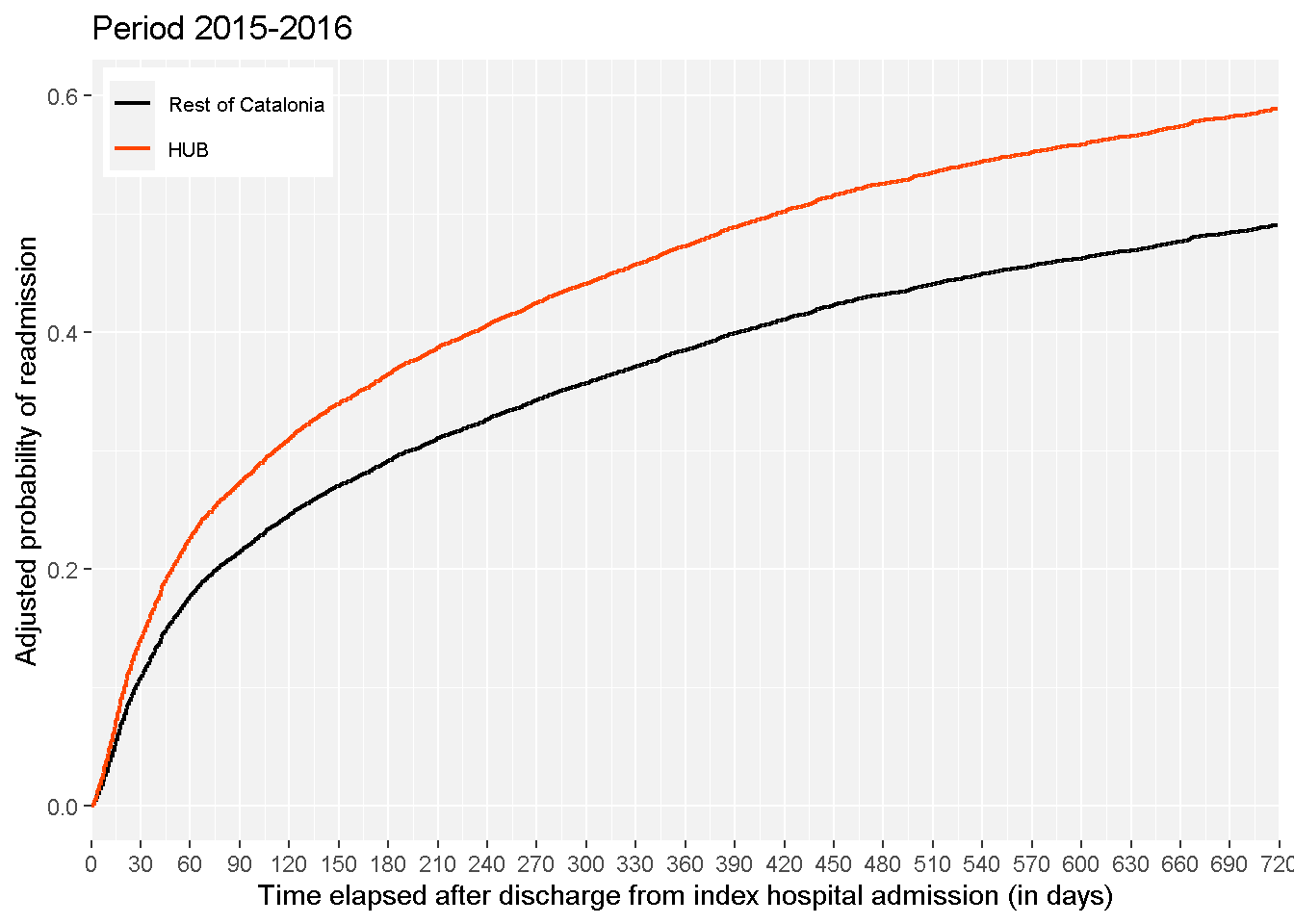


B)


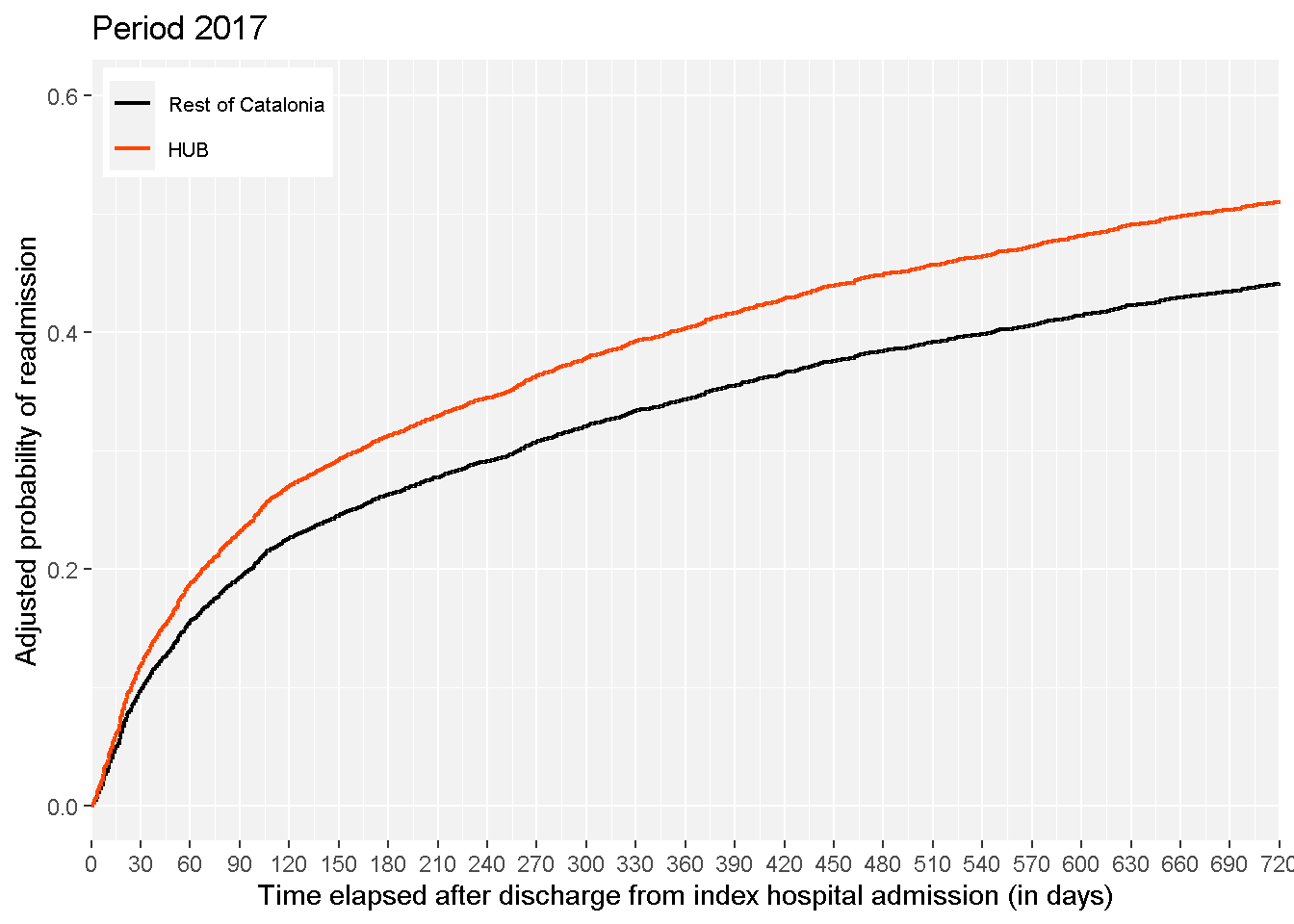


C)


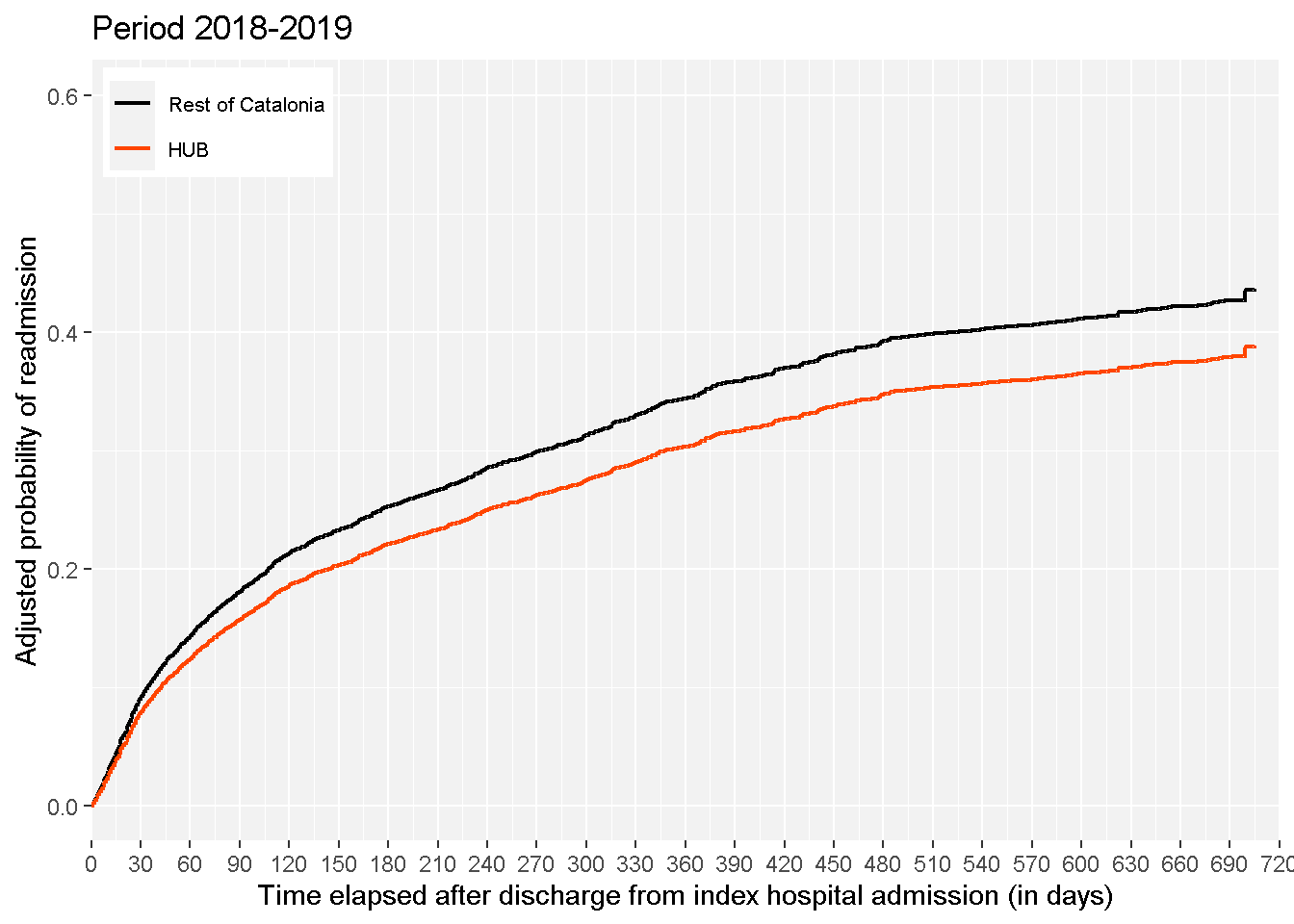


**Supplementary Figure S8.** Survival curves estimated on the basis of multivariate (adjusted) Cox models evaluating the impact on adjusted probability of HF readmission according to healthcare setting (HUB-Delta vs. Rest of Catalonia) across predefined periods: 2015-2016 (panel A), 2017 (panel B) and 2018-2019 (panel C) in the age group 75-84.

A)


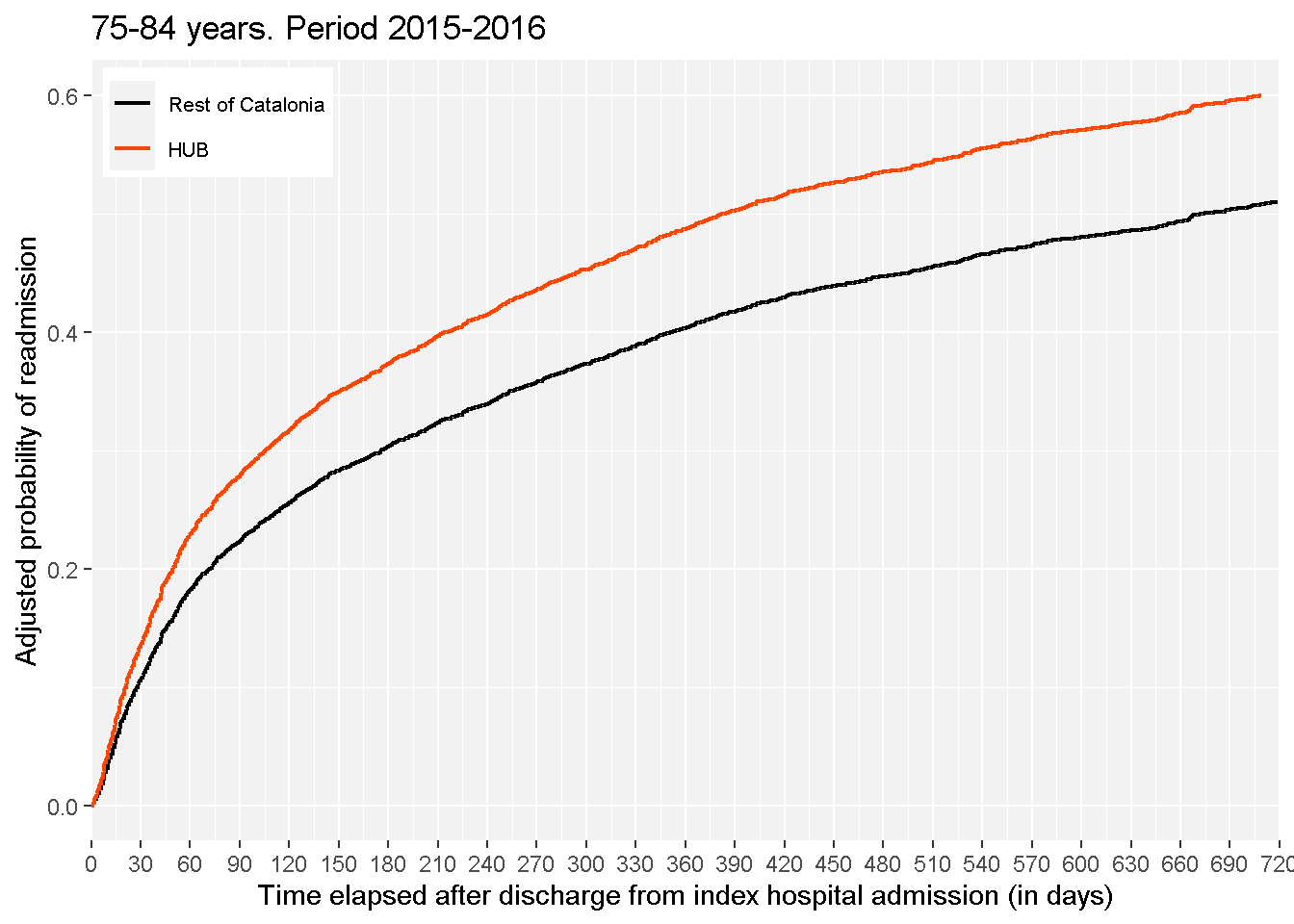


B)


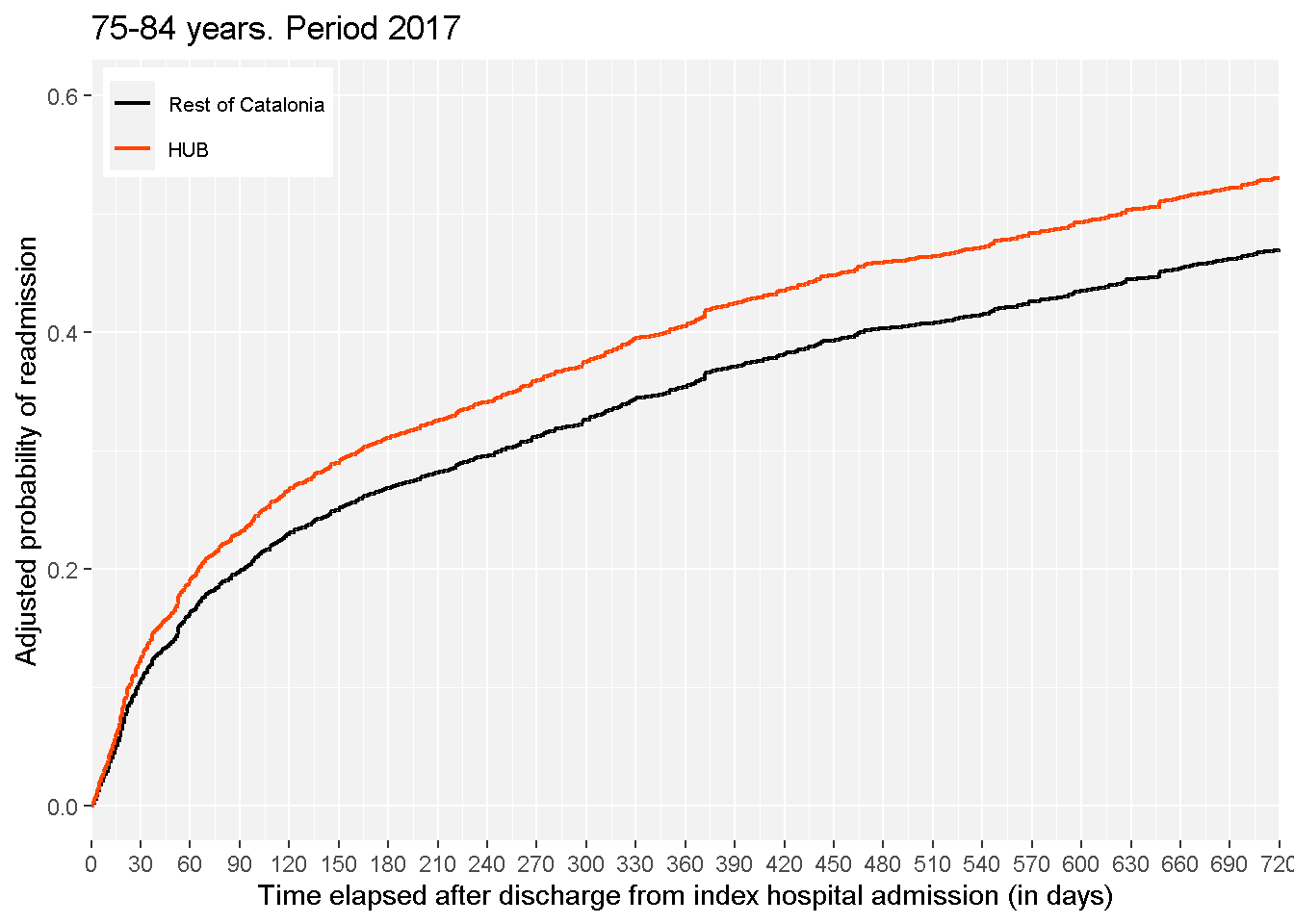


C)


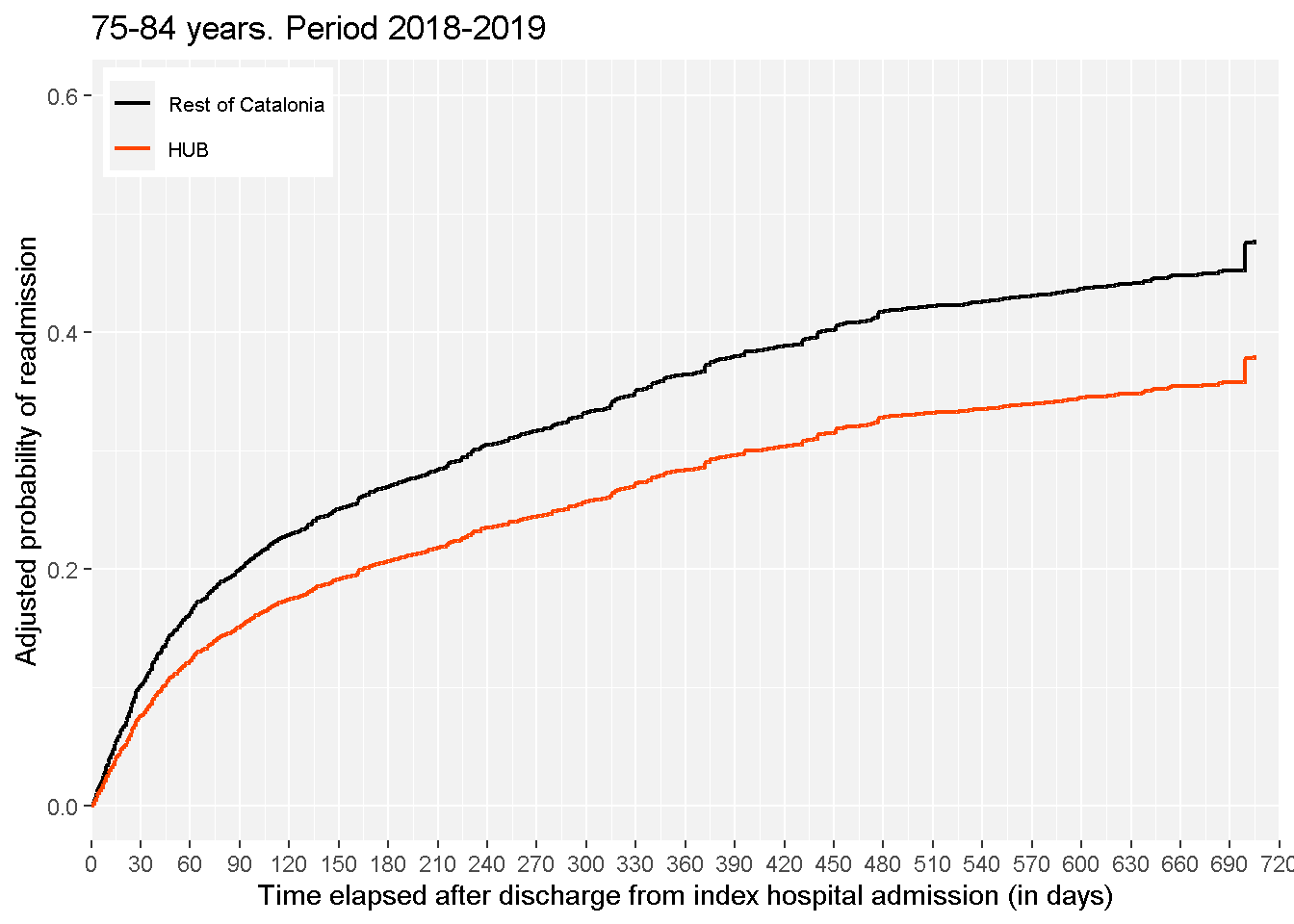


**Supplementary Figure S9.** Survival curves estimated on the basis of multivariate (adjusted) Cox models evaluating the impact on adjusted probability of HF readmission according to healthcare setting (HUB-Delta vs. Rest of Catalonia) across predefined periods: 2015-2016 (panel A), 2017 (panel B) and 2018-2019 (panel C) in the age group >84.

A)


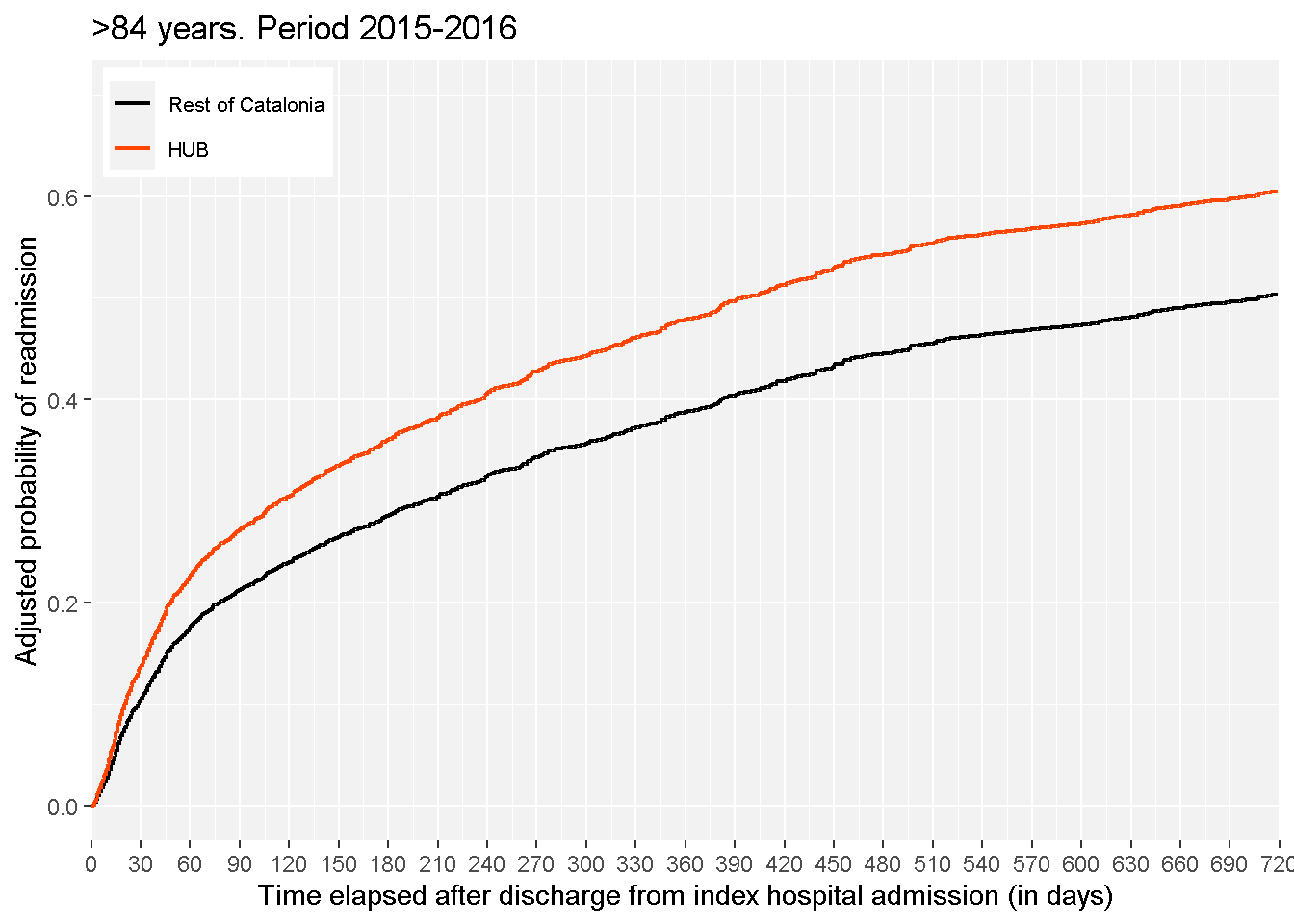


B)


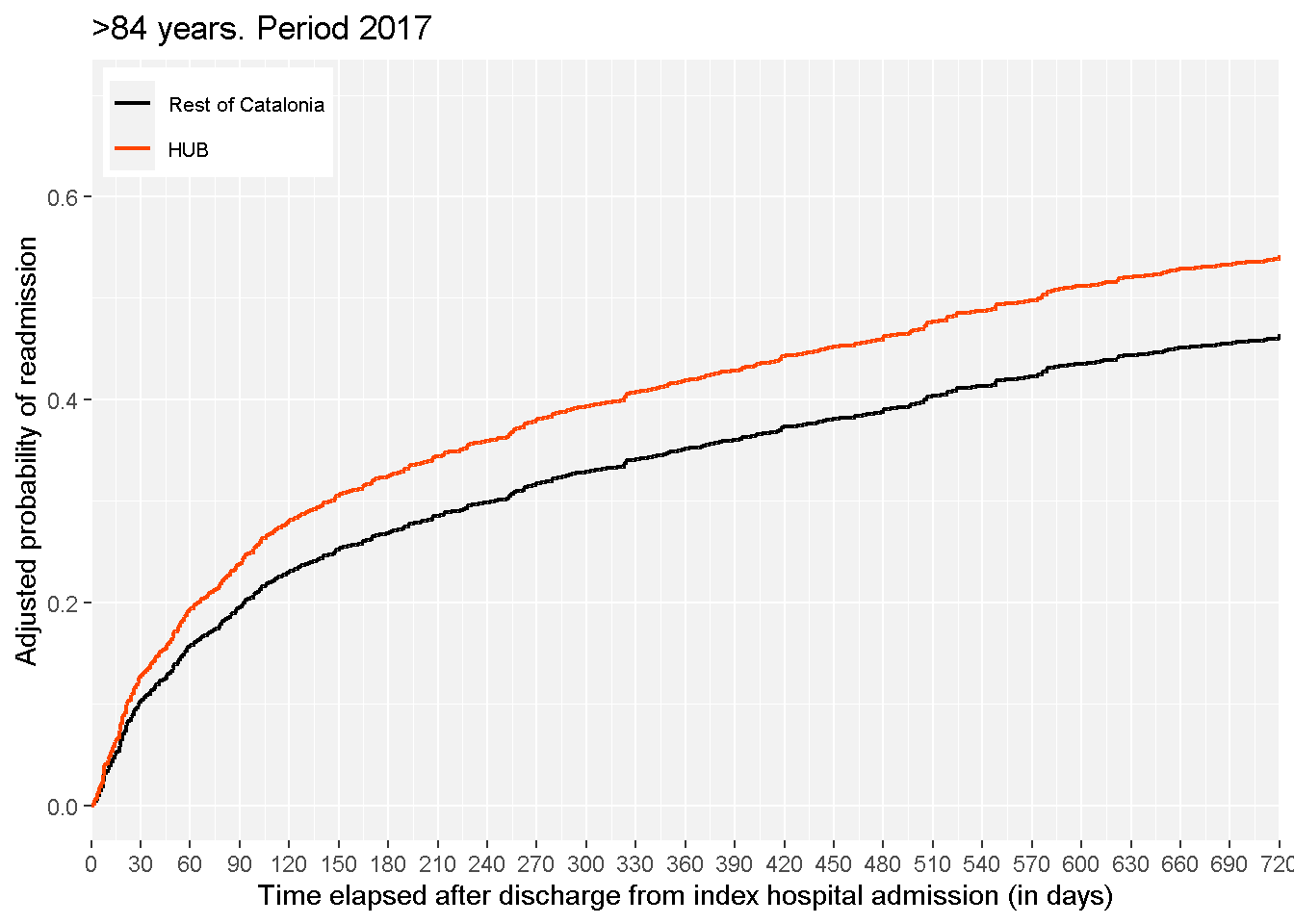


C)


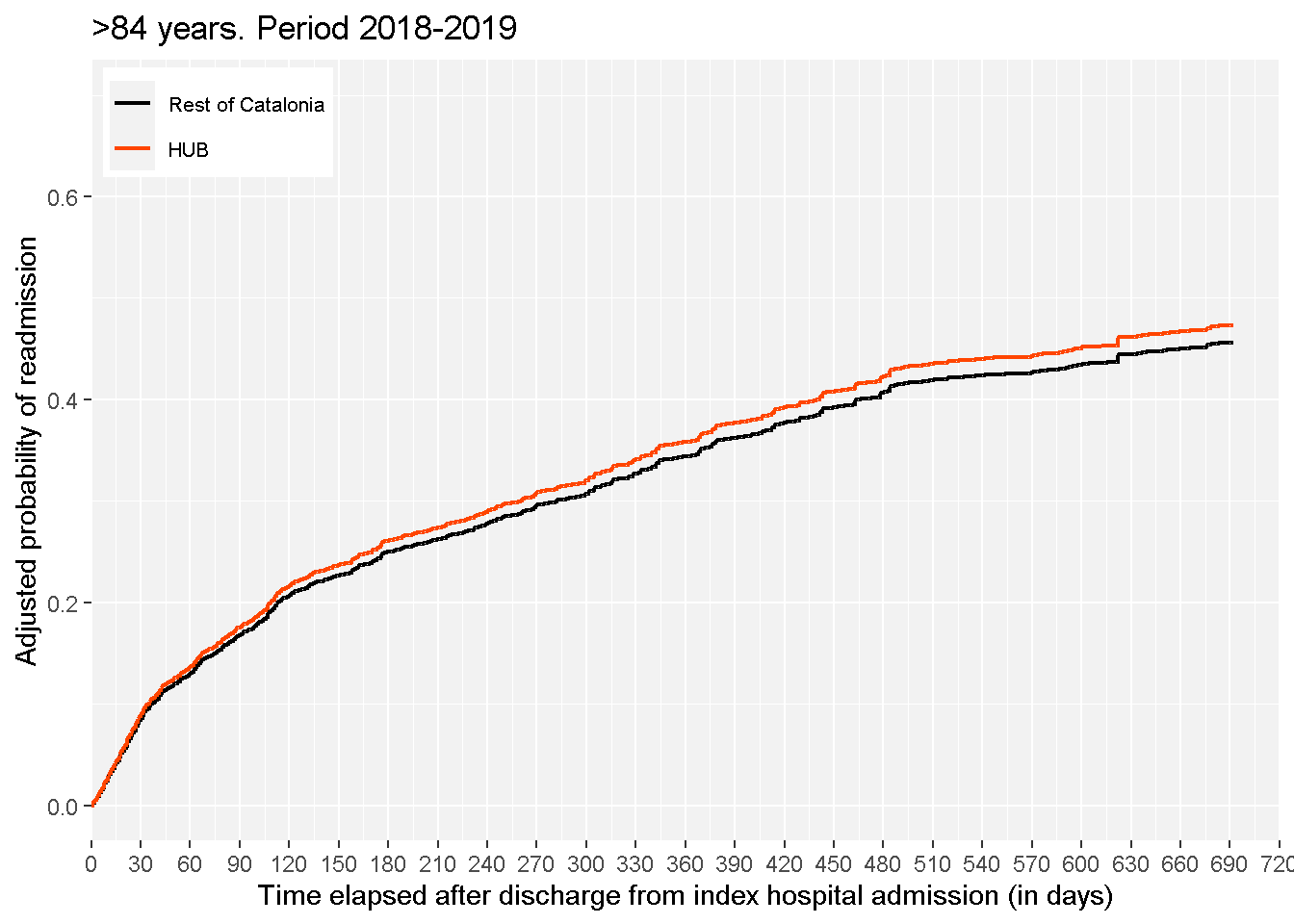

Supplement: Supplementary file 1 — Supplementary materials are available in the document Supplementary Appendix for Complementary Data and Materials. Table S1. Multivariate (adjusted) Cox proportional hazards analyses exploring the effect on outcomes of the implementation of the primary‐care hospital integrated heart failure programme according to age group strata in the HUB‐Delta healthcare area between 1 January 2015 and 31 December 2019. Models were adjusted for sex, SES, previous hospitalisation, morbidity index (GMA: associated morbidity groups) and time since diagnosis of HF. Figure S1. Survival curves were estimated on the basis of multivariate (adjusted) Cox models evaluating the impact on adjusted probability of all‐cause mortality according to healthcare setting (HUB‐Delta vs. rest of Catalonia) across predefined periods: 2015–2016 (Panel A), 2017 (Panel B) and 2018–2019 (Panel C) in the age group 15–74. Figure S2. Survival curves were estimated on the basis of multivariate (adjusted) Cox models evaluating the impact on adjusted probability of all‐cause mortality according to healthcare setting (HUB‐Delta vs. rest of Catalonia) across predefined periods: 2015–2016 (Panel A), 2017 (Panel B) and 2018–2019 (Panel C) in the age group 75–84. Figure S3. Survival curves were estimated on the basis of multivariate (adjusted) Cox models evaluating the impact on adjusted probability of all‐cause mortality according to healthcare setting (HUB‐Delta vs. rest of Catalonia) across predefined periods: 2015–2016 (Panel A), 2017 (Panel B) and 2018–2019 (Panel C) in the age group >84. Figure S4. Survival curves were estimated on the basis of multivariate (adjusted) Cox models evaluating the impact on adjusted probability of clinically related readmission according to healthcare setting (HUB‐Delta vs. rest of Catalonia) across predefined periods: 2015–2016 (Panel A), 2017 (Panel B) and 2018–2019 (Panel C) in the age group 15–74. Figure S5. Survival curves were estimated on the basis of multivariate (adjusted) [file JOIM-300-345-s001.docx]
